# Sprayed PAA-CaO_2_ nanoparticles combined with calcium ions and reactive oxygen species for antibacterial and wound healing

**DOI:** 10.1093/rb/rbad071

**Published:** 2023-08-21

**Authors:** Hong Yu, Jiale Sun, Kepeng She, Mingqi Lv, Yiqiao Zhang, Yawen Xiao, Yangkun Liu, Changhao Han, Xinyue Xu, Shuqing Yang, Guixue Wang, Guangchao Zang

**Affiliations:** Tissue and Cell Biology, Lab Teaching & Management Center, Chongqing Medical University, Chongqing 400016, China; Tissue and Cell Biology, Lab Teaching & Management Center, Chongqing Medical University, Chongqing 400016, China; Tissue and Cell Biology, Lab Teaching & Management Center, Chongqing Medical University, Chongqing 400016, China; Tissue and Cell Biology, Lab Teaching & Management Center, Chongqing Medical University, Chongqing 400016, China; Tissue and Cell Biology, Lab Teaching & Management Center, Chongqing Medical University, Chongqing 400016, China; Tissue and Cell Biology, Lab Teaching & Management Center, Chongqing Medical University, Chongqing 400016, China; Tissue and Cell Biology, Lab Teaching & Management Center, Chongqing Medical University, Chongqing 400016, China; Tissue and Cell Biology, Lab Teaching & Management Center, Chongqing Medical University, Chongqing 400016, China; Tissue and Cell Biology, Lab Teaching & Management Center, Chongqing Medical University, Chongqing 400016, China; Chongqing University Central Hospital, Chongqing Emergency Medical Center, Chongqing 400030, China; Key Laboratory for Biorheological Science and Technology of Ministry of Education, State and Local Joint Engineering Laboratory for Vascular Implants, Bioengineering College of Chongqing University, Chongqing 400030, China; Jinfeng Laboratory, Chongqing 401329, China; Tissue and Cell Biology, Lab Teaching & Management Center, Chongqing Medical University, Chongqing 400016, China; Key Laboratory for Biorheological Science and Technology of Ministry of Education, State and Local Joint Engineering Laboratory for Vascular Implants, Bioengineering College of Chongqing University, Chongqing 400030, China; Jinfeng Laboratory, Chongqing 401329, China

**Keywords:** polyacrylic acid, calcium peroxide, nanomedicines, antibacterial, wound healing

## Abstract

The most common socioeconomic healthcare issues in clinical are burns, surgical incisions and other skin injuries. Skin lesion healing can be achieved with nanomedicines and other drug application techniques. This study developed a nano-spray based on cross-linked amorphous calcium peroxide (CaO_2_) nanoparticles of polyacrylic acid (PAA) for treating skin wounds (PAA-CaO_2_ nanoparticles). CaO_2_ serves as a ‘drug’ precursor, steadily and continuously releasing calcium ions (Ca^2+^) and hydrogen peroxide (H_2_O_2_) under mildly acidic conditions, while PAA-CaO_2_ nanoparticles exhibited good spray behavior in aqueous form. Tests demonstrated that PAA-CaO_2_ nanoparticles exhibited low cytotoxicity and allowed L929 cells proliferation and migration *in vitro*. The effectiveness of PAA-CaO_2_ nanoparticles in promoting wound healing and inhibiting bacterial growth *in vivo* was assessed in SD rats using full-thickness skin defect and *Staphylococcus aureus* (*S.aureus*)-infected wound models based thereon. The results revealed that PAA-CaO_2_ nanoparticles demonstrated significant advantages in both aspects. Notably, the infected rats’ skin defects healed in 12 days. The benefits are linked to the functional role of Ca^2+^ coalesces with H_2_O_2_ as known antibacterial and healing-promoted agents. Therefore, we developed nanoscale PAA-CaO_2_ sprays to prevent bacterial development and heal skin lesions.

## Graphical abstract

**Figure:**
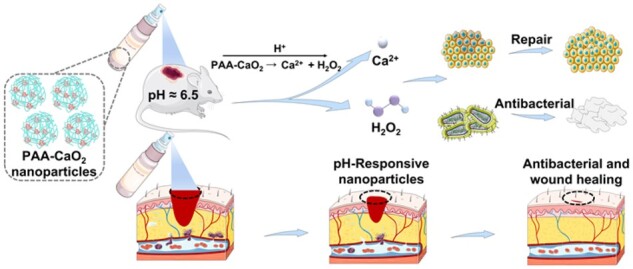

## Introduction

Skin is the most significant physical barrier against external pathogens. Nevertheless, skin damage can result from surgical removal of tumors, diabetic ulcers and unintentional wounds. If skin injuries are left untreated, microbial colonization may grow, which could lead to disability or even death [1, 2]. The intrinsic stages of wound repair involve hemostasis, inflammation, proliferation and remodeling [3]. Delay in recovery raises severe concerns about the necessity for additional therapeutic interventions. The patients must take antibiotics to prevent infection until their skin regains its barrier function. Therefore, there is always a risk of infection during the different stages of wound-healing therapy [4]. Furthermore, implementing autologous or allogeneic transplantation is constrained by the limited supply of donors and the high cost, even in clinically prevalent flap transplant therapy. An alternate method for stimulating skin repair at the defect sites is wound dressings. To promote skin healing, fibrous membranes and hydrogel-style wound dressings have been extensively researched [5, 6]. Nevertheless, fiber pads are easily separated from wounds, which could significantly lengthen the time before full ingrowth. Soft hydrogels can fill irregular wounds, yet it impedes gas exchange at the wound site. Therefore, new wound dressing compositions are urgently needed to address the shortcomings.

Recently, Zhang *et al*. reported the synthesis of SH-CaO_2_ nanoparticles demonstrating antitumor therapy. They showed that the SH-CaO_2_ nanoparticles decomposed into Ca^2+^ and H_2_O_2_ under weakly acidic conditions (pH 6.5), appropriate for the tumor microenvironment [7]. H_2_O_2_, a reactive oxygen species (ROS) family member, would lead to an effective antitumor activity synergistic calcium overload effect. The process of repairing skin injury involves numerous cells and molecular ions. Among them, Ca^2+^ is involved in several signaling cascades as a critical secondary messenger regulating wound healing [8]. The mechanism illustrates that Ca^2+^ can modulate inflammatory cell infiltration, fibroblast proliferation, migration and differentiation, simultaneously enhancing angiogenesis. Moreover, Ca^2+^ acts as a stationary part in maintaining physiological hemostasis. Ca^2+^-incorporated nanofiber films have been employed for wound dressing, demonstrating improved cell proliferation, antibacterial activity and subsequent wound healing [9–11].

When skin damage occurs, H_2_O_2_ is elevated in the surrounding tissues and accumulates before it gradually declines. The change in H_2_O_2_ dynamic levels accompanies wound healing, as the concentration of H_2_O_2_ in the wounds affects trauma recovery to a certain degree. The signified H_2_O_2_ roles function as a signaling molecule or secondary messenger like Ca^2+^, transmitting stress messages and stimulating effector cells to respond [12, 13]. On the other hand, H_2_O_2_ acts as an antimicrobial agent and has been widely used for controlling bacterial reproduction [14, 15].

Inspired by these innovative studies, we considered whether nanoparticles with internal CaO_2_ cores could enhance cell proliferation and inhibit bacteria growth. However, solitary CaO_2_ nanoparticles have the possibility of aggregation [16]. To prevent CaO_2_ nanoparticles from congregating and control their particle size or morphology [17], we introduced PAA as a surface modifier to synthesize amorphous CaO_2_ nanoparticles (PAA-CaO_2_ nanoparticles) that can be applied in curing full-thickness skin defects.

It is reported that the pH of wound sites gradually shifts to an acidic level during the wound-healing process. Local acidification is induced by lactic or acetic acid production by colonized bacteria strains [18]. PAA-CaO_2_ nanoparticles will be decomposed into Ca^2+^ and H_2_O_2_ under a pathological acidic (pH 6.5) wound microenvironment steadily and continuously, which is analogous to a previous report [19]. The released Ca^2+^ and H_2_O_2_ are potent molecules for impeding bacterial survival. They significantly heightened the levels of calcium, PI3K/AKT and MEK1/2/ERK1/2 signaling pathway-related molecules such as PLC-δ4, PI3K and ERK1/2. These effects not only facilitate antibacterial endeavors but also foster angiogenesis, cell proliferation and migration [20]. Benefiting from the multiple functions, PAA-CaO_2_ nanoparticles realize the therapeutic effects on skin wounds.

**Figure 1.**
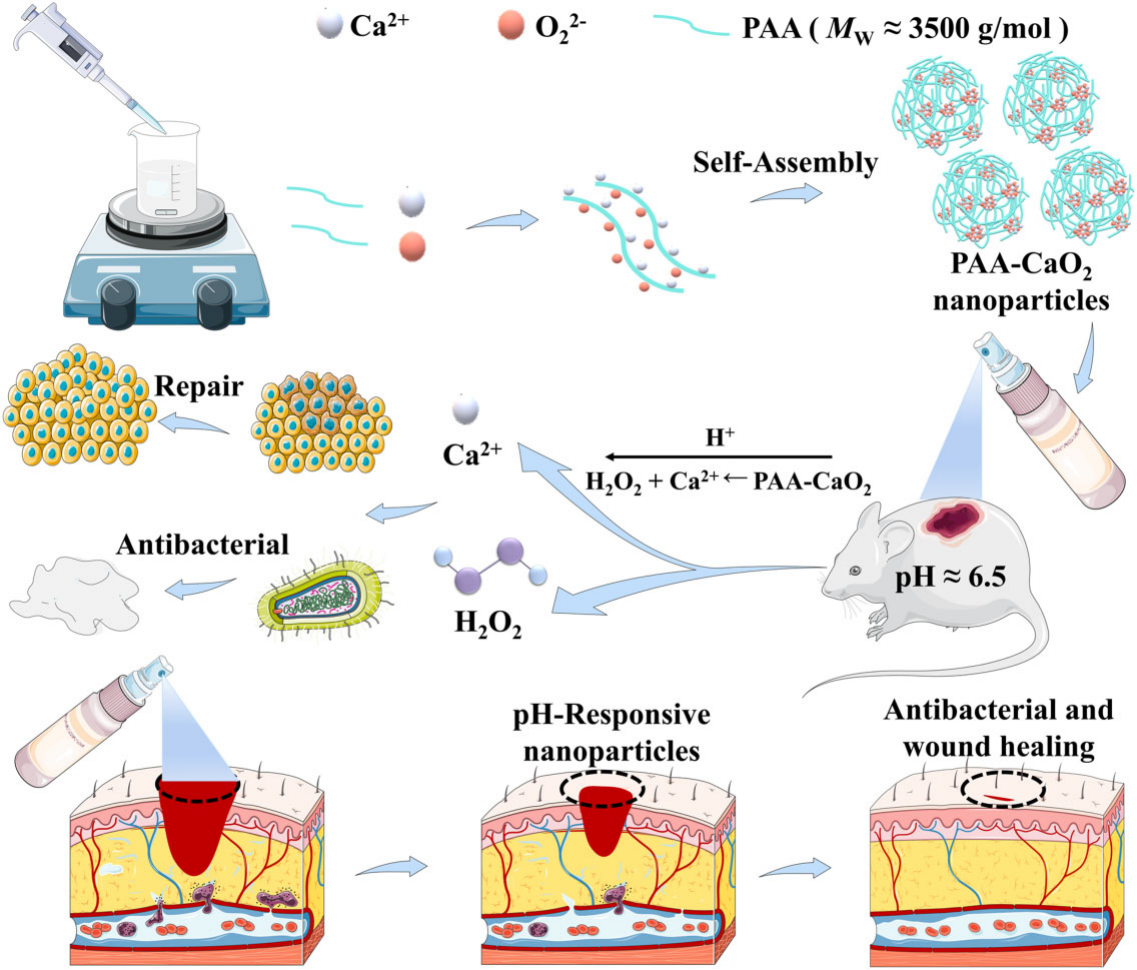
Schematic illustration of PAA-CaO_2_ nanoparticles for accelerating wound healing.

As shown in Fig. 1, PAA-CaO_2_ nanoparticles decomposed into Ca^2+^ and H_2_O_2_ in the wound acidic surroundings after spraying on the full-thickness skin defect rats. Ca^2+^ coalesces with H_2_O_2_, effectively maintaining anti-infection wound status and regulating wound healing. PAA-CaO_2_ nanoparticles promote fibroblast proliferation and accelerate wound regeneration by contributing to the antimicrobial and appropriate inflammatory response. PAA-CaO_2_ nanoparticles were prepared to validate this concept, and their acidic-triggered decomposition was confirmed *in vitro*. After that, the *in vitro* antibacterial effects of PAA-CaO_2_ nanoparticles were evaluated by employing two typical bacterial strains commonly parasitized in skin wounds. The L929 cells were used for *in vitro* cell proliferation and migration investigation. Wound-healing efficacy of PAA-CaO_2_ nanoparticles was assessed in SD rats using a full-thickness skin defect model (general wounds) *in vivo*. Further application in the *S.aureus*-infected wounds (infected wounds) was studied. Overall, this research aimed to provide evidence for the wound-healing catalytic properties of PAA-CaO_2_ nanoparticles in different wound-healing sprays.

## Experiment section

### Synthesis of PAA-CaO_2_ nanoparticles

PAA-CaO_2_ nanoparticles were fabricated according to the previous report [11]. Briefly, CaCl_2_ (100 μl, 1 M) and PAA (100 μl, 0.5 mg/ml) were mixed in the 10 ml absolute ethanol solution under magnetic stirring at room temperature. After that, the above solution was added in NH_3_·H_2_O (300 μl, 5%) and H_2_O_2_ (10 μl, 30%) sequentially. After stirring for 10 min, the formed nanoparticles were collected by ultrafiltration, centrifugation and washed with methanol at least three times. The obtained PAA-CaO_2_ nanoparticles were then dispersed in ultrapure water to configure as 1 mg/ml nano-spray for study. To obtain unmodified CaO_2_ nanoparticles, the PAA solution was replaced by deionized water in the above procedure.

### Characterization

The morphology of PAA-CaO_2_ nanoparticles was observed using scanning electron microscopy (SEM, SU8010, Hitachi, Ltd, Japan) after freeze-drying, and SEM determined the EDS mapping images with a working voltage of 3 kV. The average size and zeta potential of PAA-CaO_2_ nanoparticles were measured with a Zetasizer-ZS90 (Malvern Instruments, Malvern, UK). Thermogravimetric analysis (TGA) was performed using a thermal gravimetric analyzer (TASDT 650, TA, USA) and the measurements were tested using the standard mode. Fourier transform infrared spectroscopy (FTIR) (Thermo Fisher Scientific-CN, USA) spectra were analyzed within the range of 4000–400/cm. Qualitative analysis of the surface elements of PAA-CaO_2_ nanoparticles was given by X-ray photoelectron spectroscopy (XPS) PHI5702, Al Kα (1486.7 eV) with monochromatic. The binding energy was calibrated using the C 1 s standard peak of 284.6 S-4eV as a reference [15, 21].

### Sprayability analysis of PAA-CaO_2_ nanoparticles

Sprayed PAA-CaO_2_ nanoparticles solution at 1 mg/ml concentration mixed with a red dye was transferred into a commercial plastic spray bottle, and the sprayability of PAA-CaO_2_ nanoparticles was then tested at room temperature [22].

### Measurement of the CaO_2_ loading capacity in PAA-CaO_2_ nanoparticles

The amount of CaO_2_ (*M*_W_ ≈ 72 g/mol) incorporated in PAA-CaO_2_ nanoparticles was determined by using an inductively coupled plasma source mass spectrometer (ICP-MS, Agilent7900, USA) to detect the concentration of Ca^2+^ in the Supernatant after the encapsulation. Firstly, 100 μl of 1M CaCl_2_ (C_0_) was added to 10 ml absolute ethanol to synthesize PAA-CaO_2_ nanoparticles, and the total synthetic system volume was calculated (V). After synthesizing PAA-CaO_2_ nanoparticles, the precipitate was obtained by centrifugation and weighed (m_PAA-CaO2 nanoparticles_) after freeze-drying. Then all the supernatants were collected, and a certain volume of hydrochloric acid (2 M) was added. The concentration of Ca^2+^ in the supernatant (C_s_) was measured by ICP-MS. The CaO_2_ loading capacity was calculated based on the following formula: CaO_2_ loading capacity (%, w/w) = the mass of CaO_2_ loaded in PAA-CaO_2_ nanoparticles/the mass of PAA-CaO_2_ nanoparticles × 100% = [(C_0_ − C_s_) × V × 72]/m_PAA-CaO2 nanoparticles_ × 100% [23].

### Acid-induced Ca^2+^ release profiles

The generation of Ca^2+^ after the decomposition of CaO_2_ nanoparticles and PAA-CaO_2_ nanoparticles was detected by ICP-MS through the bag filter method. Dialysis bags loaded with 2 ml of 1 mg/ml CaO_2_ nanoparticles or PAA-CaO_2_ nanoparticles were immersed in 30 ml PBS (calcium-free solution) with different pH values of 6.5 and 7.4 to mimic the environment of chronic wound (pH 6.5) and normal tissues/blood (pH 7.4), respectively [24]. All the samples were oscillated at 100 rpm in a thermostat at 37°C. At varied time points, a 4-ml buffer sample was taken to examine the Ca^2+^ concentration and replaced with an equal volume of fresh medium. The percentage of released Ca^2+^ was then calculated [7].

### Blood clotting test

A blood clotting test was used to evaluate the effect of PAA-CaO_2_ nanoparticles on blood clotting. Briefly, 0.5 ml of 1 mg/ml PAA-CaO_2_ nanoparticles suspension in PBS at pH 7.4 and 6.5 and isometric anticoagulated rabbit whole blood were mixed in a citrate anticoagulated tube, respectively. Each sample was time-recorded and photographed [25].

### Hemocompatibility of PAA-CaO_2_ nanoparticles

The hemocompatibility of PAA-CaO_2_ nanoparticles was investigated by a hemolysis test. Fresh rabbit blood was centrifuged at 1500 rpm to isolate RBCs. Purified RBCs were diluted with PBS to obtain RBC suspension (2%, v/v). One milliliter RBCs suspension mixed with 20 µl PBS as a negative control, 20 µl 0.1% Triton X-100 as a positive control and 20 µl different materials as experimental groups. After being incubated at 37°C for 1 h, the mixture in tubes was centrifuged at 1500 rpm for 15 min. A microplate reader was used to measure the absorbance of the supernatant. OD_t_, OD_n_ and OD_p_ are the absorbance values of the experimental groups, negative control (PBS) and positive control (1% Triton-X), respectively. HR(%) = [(OD_t_ − OD_n_)/(OD_p_ − OD_n_)] × 100% [15, 26].

### *In vitro* anti-infection properties of PAA-CaO_2_ nanoparticles

Gram-positive (*S.aureus*) and Gram-negative (*Escherichia coli* (*E.coli*)) bacteria were used in bacterial experiments. A modified disc diffusion test (K-B) method was used for the antibacterial activity of normal saline (control), CaO_2_ and PAA-CaO_2_ nanoparticles. The bacterial solution was prepared by inoculating *S.aureus* and *E.coli* in Luria–Bertani (LB) liquid medium and oscillating at 37°C (100 rpm) overnight. It was inoculated on agar plates and divided into three groups. Place 6 mm filter paper discs wholly immersed in two spray solutions and normal saline in the center of the plate. After incubation at 37°C for 24 h, the area of the antibacterial ring was measured to evaluate the antibacterial ability [19, 27].

To obtain SEM images of bacteria, bacteria (1 × 10^9^ CFU/ml) were harvested via centrifugation at 12 000 rpm for 3 min, then dispersed to PBS (pH 7.4 and 6.5) and cultured at 37°C for 2 h. After that, bacteria were collected by centrifugation and fixed with 2.5% glutaraldehyde for 4 h at 4°C. The bacteria were rinsed with PBS (pH 7.4) and dehydrated using ethanol. Finally, samples were observed under SEM [15].

### *In vitro* pH-dependent ROS generation

Taking advantage of the fact that PAA-CaO_2_ nanoparticles could be decomposed into Ca^2+^ and H_2_O_2_ in an acidic environment. Since the latter is a type of ROS, which might be attributed to the antibacterial effect of PAA-CaO_2_ nanoparticles. Therefore, we examined the generation of ROS in bacteria after treatment with PAA-CaO_2_ nanoparticles using DCFH-DA fluorescent probe. Briefly, 1 ml *E.coli* and *S.aureus* (10^8^ CFU/ml) were exposed to PBS (pH 7.4 and pH 6.5) after collected by centrifugation, followed by added normal saline (control), CaO_2_ and PAA-CaO_2_ nanoparticles cultured for 4 h at 37°C with shaking gently. Then, the bacteria were centrifuged at 12 000 rpm for 3 min, and DCFH-DA (10 μM) was sequentially mixed with bacteria solutions and stained in the dark for 30 min. Finally, the bacteria were rinsed with PBS, and samples were photographed under fluorescence microscopy [7].

### Cytotoxicity assay

Fibroblast (L929 cell line) was chosen as the model cell in this study because it is closely associated with wound healing [28]. CCK-8 assay was employed to investigate *in vitro* cytotoxicity of CaO_2_ and PAA-CaO_2_ nanoparticles. L929 cells were cultured in 96-well plates (5 × 10^3^ cells/ml) and incubated for 12 h to make the cells adherent. Then, cells of each well were washed with PBS and further incubated in a fresh medium containing 10 μl of different concentration series of spray solutions and normal saline as the control for 24 h. Subsequently, the medium was discarded, the plates were washed with PBS, the culture medium containing 10% CCK-8 was added to each well, and the cells were incubated at 37°C for 2 h. The optical density was determined at a wavelength of 450 nm with a spectrophotometer (Thermo Fisher Scientific-CN, USA) to assess cytotoxicity and cell viability [19].

### Cell proliferation assay

Actin staining was carried out to perform the cell proliferation assay. After seeding L929 cells in six-well plates for 24 h, the original culture medium was replaced with a fresh medium containing normal saline (control), CaO_2_ and PAA-CaO_2_ nanoparticles for another 24 h. Then the medium was discarded, and the plates were washed with PBS. Three hundred microliters of 4% formaldehyde solution were added to each well to fixed cells for 15 min. Next, cells were washed using a shaker (Kylin-Bell Lab Instruments Co., Ltd, 5 min, 50 rpm). Three hundred microliters of 0.2% Triton X-100 solution were added to each well to permeabilization for 15 min. Then, cells were washed with PBS. After that, 600 μl of 3% BSA was added to each well for 1 h. Then 300 μl of diluted FITC-Phalloidin was added and incubated in the dark for 15 min to visualize the cytoskeleton. Finally, 300 μl DAPI staining solution was added to counterstain for 30 s to stain the cell nuclei. The images were observed under a fluorescence microscope (Nikon A1+R-980 confocal microscope).

### Cell migration assay

L929 cells were seeded in 6-well plates (5 × 10^5^ cells/ml) and incubated at 37°C for the cell migration assay. After the confluent monolayers were formed in each well, a sterile pipet tip (200 μl) made a straight scratch. Then the cells were incubated in a serum-free medium containing CaO_2_, PAA-CaO_2_ nanoparticles or the same amount of normal saline as the control for 24 h. The scratches were photographed using a digital camera-equipped inverted microscope for statistical analysis. Image J software analyzed the cell migration rate.

### Intracellular ROS and Ca^2+^ detection assays

DCFH-DA and Fluo-4, AM were employed as fluorescent ROS and Ca^2+^ probes to indicate CaO_2_ and PAA-CaO_2_ nanoparticles induced ROS and Ca^2+^ generation. Briefly, L929 cells were exposed to normal saline (control), CaO_2_ and PAA-CaO_2_ nanoparticles for 4 h, respectively. Then, the cells were stained with DCFH-DA and Fluo-4, AM.

For DCFH-DA staining: L929 cells were incubated in the 24-well plates for 12 h to adhere. Then 50 μl of the stimulator (CaO_2_ or PAA-CaO_2_ nanoparticles or normal saline as control) and 150 μl of DAPI solution were added to each well. After 12 h of incubation at 37°C, the original medium was discarded, and the cells were washed using PBS on a shaker. Next, 300 μl of DCFH-DA (10 mM) was added to each well and co-cultured at 37°C for 40 min. Finally, the probe was discarded, and the cells were incubated in DMEM for 20 min at 37°C to fully hydrolyze the probe and washed for further confocal laser scanning microscopy (CLSM) observation and analysis.

For Fluo-4, AM staining: L929 cells were pretreated before staining following the same procedure as above. Then the medium was removed, and the Fluo-4, AM solution was added to each well. Next, the cells were washed to adequately remove residual Fluo-4, AM stain. PBS was added to cover the cells to ensure complete de-esterification of the AM group in the cells. The cells were examined by fluorescence microscopy [7].

### General wound healing assay

The experimental scheme and operation have been approved by the Ethics Committee of Chongqing Medical University, and the animals in the experiment are under human care. Adult male SD rats (6–8 weeks) were used as experimental animals. The dorsal surface hairs were shaved after anesthesia with an isoflurane volatile anesthesia machine (RWD Life Science, Shenzhen, China). A circle wound (D = 1 cm) was removed from the back of the skin by a circular perforator. Wounds in all groups were disinfected with iodophor and alcohol. The rats were randomly divided into four groups. All groups were disinfected every 2 days, then the normal saline treated-group as control, the CaO_2_ and PAA-CaO_2_ nanoparticles group was treated with corresponding sprays, and the SS was treated with sulfadiazine silver cream (SS). The wound areas were photographed every 7 days and calculated by Image J software.

### Transcriptomic analysis and western blot assay

Skin wound samples from SD rats on Day 7 were used for RNA sequence (RNA-seq) and western blotting (WB), because the wound healing processes were in the proliferative phase and neovascularization was evident on Day 7 [3].

For transcriptomic analysis: the wounds were treated with normal saline or PAA-CaO_2_ nanoparticles for 7 days. Subsequently, differentially expressed genes (DEGs) were determined by DESeq2. DEGs were used for heatmap analysis using bioinformatics (https://www.bioinformatics.com.cn/). Gene set enrichment analysis, including Gene Ontology (GO) and Kyoto Encyclopedia of Genes and Genomes (KEGG), was conducted using DEGS with clusterProfiler. GO analysis was visualized by the GO plot.

For western blot assay: after grinding the wound tissue at low temperature, it was further treated at 4°C with a glass homogenizer. The supernatant was collected by centrifugation at 700×g (ICEN-24R) for 10 min at a temperature of 4°C and centrifuged again at 14000×g for 30 min to precipitate cell membrane debris. Finally, cytoplasmic proteins were collected. A bicinchoninic acid assay reagent kit was used to determine protein concentration. The expression level of the PLC-δ4, PI3K, AKT, MEK1/2, ERK1/2 and VEGF of wound skin tissue after incubation with different formulas were detected by WB to study antibacterial and wound healing mechanisms. Finally, the signal density of the bands was visualized via the MiniChemi Mini Size Chemiluminescent Imaging System (Beijing Sage Creation Science Co., Ltd) and quantified by ImageJ software.

### *Staphylococcus aureus*-infected wound healing assay

A full-thickness skin defect model of infection was established to evaluate the antibacterial ability of PAA-CaO_2_ nanoparticles further. Because CaO_2_ nanoparticles spray had no superiority in the healing effect of the general wound since the rats were only randomly divided into three groups in this test: control (normal saline), SS and PAA-CaO_2_ to compare the therapeutic efficacy of PAA-CaO_2_ nanoparticles and commercial SS cream on *S.aureus*-infected wounds. SD rats (6–8 weeks) were used to establish an injury model. The back of the mouse was incised, and 100 µl of 1 × 10^6^ CFU/ml *S.aureus* suspension was injected. During the treatment, bacteria were counted in exudate using the plate dilution method in the first 3 days. Briefly, 12 h after the infection models were established, three rats were randomly selected from each group. After the swab was used to wipe the infected wound exudate, it was immersed in 3 ml of normal saline for 12 h. The solution was coated on agar plates by spread plate method and then incubated at 37°C for 24 h. Dilute the bacteria 10^4^ times before plating for the 12 h-infected. Day 1 and 3 bacteria were diluted 10^2^ times before plating. The bacterial count was then performed by Image J software [29]. Materials were given once a day on the wound in corresponding groups. The wound areas were photographed every 4 days and calculated by Image J software.

### Histological analysis

The rats in the general wound group were sacrificed on 3, 7, 14 and 21 days, and the rats in the infected wound group were sacrificed on 3, 7 and 12 days, respectively. The wound skin was taken for tissue staining to observe wound healing. The rats’ organs (heart, liver, spleen, lung, kidney, brain, testis) were obtained for staining to study the pilot toxicity of PAA-CaO_2_ nanoparticles. After fixing the tissue in 4% paraformaldehyde for 3 days, the tissue was embedded in paraffin by dehydration. After the embedded tissue was sectioned into 2 μm sections with a micro-chipper and tiled on adhesive glass slides, the slices were incubated overnight at 50°C. Hematoxylin and eosin (H&E) staining of skin tissues was performed to observe the size of the wound bed, and Masson’s trichrome staining was performed to observe collagen deposition. H&E stained the organs sections images were observed and quantified using a confocal fluorescence microscope and Image J software.

Immunofluorescence (IF) staining of CD68, CD31 and proliferating cell nuclear antigen (PCNA) in general wounds was also studied. Tissue sections were treated with 1× EDTA antigen repair solution for antigen extraction, and endogenous HRP enzyme was blocked with 3% H_2_O_2_. After blocking with 10% normal goat serum, primary antibodies CD68, PCNA, CD31 and secondary antibodies HRP labeled sheep anti-rabbit was added for incubation, respectively. The multiple fluorescence immunohistochemistry kit was used for chromogenic staining. Finally, the images were taken under a fluorescence microscope.

The details regarding reagents used are provided in the Supplementary material.

## Results and discussion

### Preparation and characterization of PAA-CaO_2_ nanoparticles

**Figure 2.**
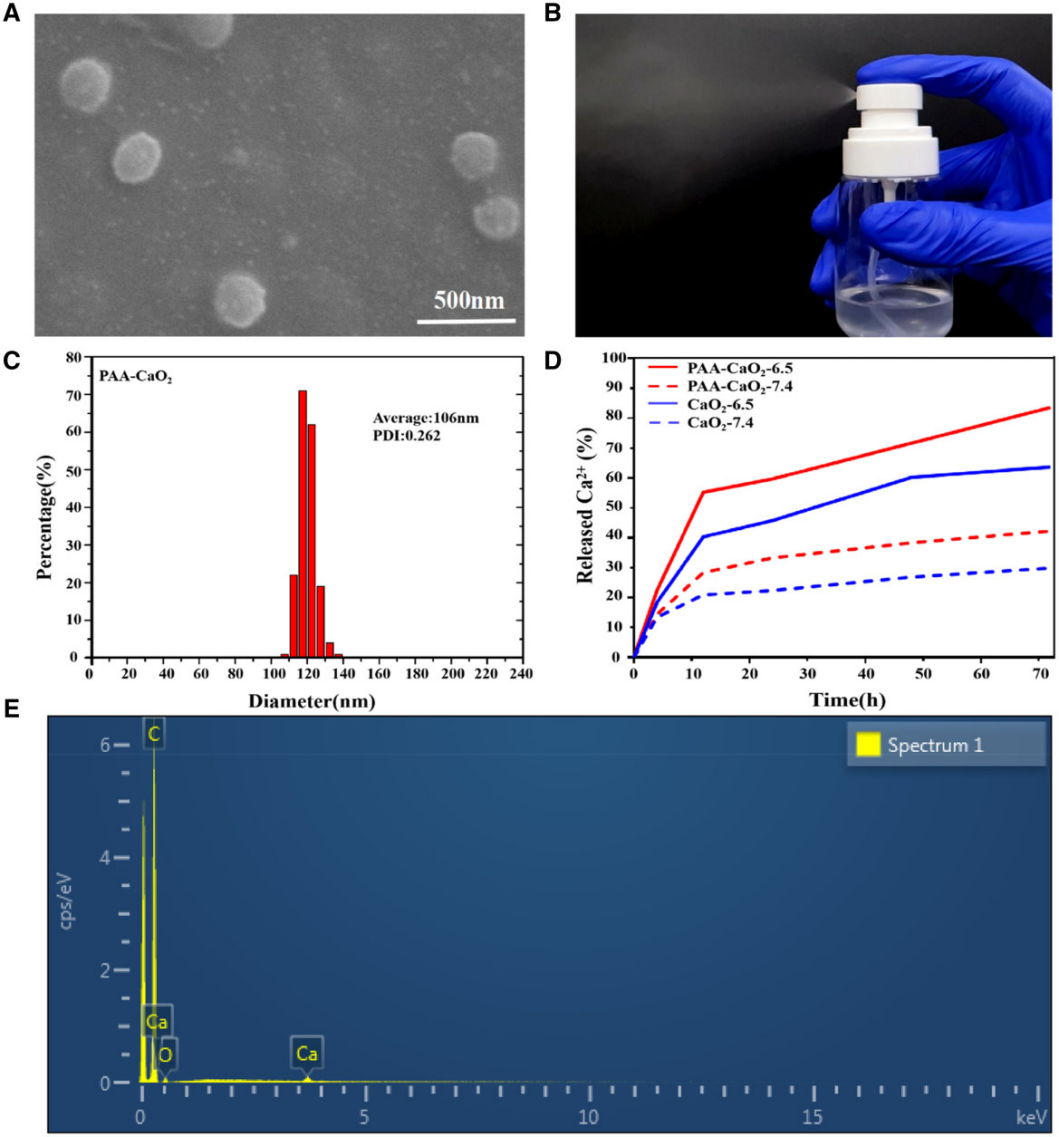
(**A**) The SEM images of PAA-CaO_2_ nanoparticles. (**B**) Re-dispersion of PAA-CaO_2_ nanoparticles in ultrapure water after lyophilization and sprayed out from a spray nozzle. (**C**) The hydrodynamic diameter of PAA-CaO_2_ nanoparticles was measured by dynamic light scattering. (**D**) Time-dependent Ca^2+^ release from CaO_2_ and PAA-CaO_2_ nanoparticles suspension at different pH values. (**E**) The EDS spectrum and element mapping of PAA-CaO_2_ nanoparticles.

Based on a previous study, PAA-CaO_2_ nanoparticles were created with a loading capacity of 44.25 ± 1% (w/w) of CaO_2_ in the nanoparticles. The SEM images showed that many ultra-small black dots represented PAA-CaO_2_ nanoparticles. Moreover, these nanoparticles were dispersive and surrounded by irregular nebulosity owing to PAA coating (Fig. 2A). The XPS high-resolution Ca 2p spectrum displayed two prominent peaks and one peak in the O1s spectrum, indicating the valence state of Ca was +2 and O was −1 (Peroxide ions, O–O) in PAA-CaO_2_ nanoparticles. Furthermore, the XPS full spectrum verified that O 1 s peaks at 532.5 eV were assigned to O–O, indicating the presence of peroxo groups (Supplementary Fig. S1) [30, 31]. Attributed to the PAA coating and small size, PAA-CaO_2_ nanoparticles could be easily dispersed in water and sprayed out from the spray nozzle (Fig. 2B; Supplementary Fig. S2). It would be convenient for the patients to apply PAA-CaO_2_ nanoparticles for wound therapy. In Supplementary Fig. S3, the zeta potential of CaO_2_ and PAA-CaO_2_ nanoparticles was depicted. The initial CaO_2_ nanoparticles displayed a zeta potential of approximately +0.24 ± 0.10 mV, while this value decreased to −6.89 ± 1.32 mV for PAA-CaO_2_ nanoparticles. This confirmed polyanionic electrolytes-PAA modified CaO_2_ nanoparticles by utilizing the attraction between negatively charged ions and positively charged nanocrystals [32]. Therefore, the PAA coating endowed the CaO_2_ nanoparticles with an electronegative property, which can effectively facilitate nucleation, prevent aggregation and enhance the biocompatibility of CaO_2_ nanoparticles [33]. Moreover, the successful modification was also indicated by the greater mass loss in TGA (Supplementary Fig. S4) [34]. The hydrodynamic diameter of PAA-CaO_2_ nanoparticles was ∼106 nm (Fig. 2C), consistent with the one displayed in the SEM images. The 3600–3200/cm broad absorption peak represented the hydrogen-bonded O–H stretching vibration in the FTIR absorption spectrum. It could be attributed to the hydrogen bonding networks in PAA-CaO_2_ nanoparticles (Supplementary Fig. S5). Moreover, the existence of peroxo groups was verified by the presence of characteristic peaks at 831, 881 and 1115/cm [35]. Ca and O elements are distributed at the surface of PAA-CaO_2_ nanoparticles is also verified by characterization using Energy dispersive spectrometer (EDS) (Fig. 2E).

### *In vitro* Ca^2+^ release and blood coagulation

We studied the breakdown of PAA-CaO_2_ nanoparticles in acidic conditions by measuring the amount of Ca^2+^ released *in vitro*. Our findings indicated that PAA-CaO_2_ nanoparticles degrade at a pH of 6.5 (Fig. 2D). The results revealed that these nanoparticles are sensitive to changes in pH, as the release of Ca^2+^ increased when the pH decreased from 7.4 to 6.5. The increased release of Ca^2+^ in acidic surroundings was attributed to the degradation of CaO_2_ and the reduction of electrostatic interactions between PAA and CaO_2_, with less deprotonation of the carboxyl group (–COOH) of PAA as the pH decreased [17]. Interestingly, PAA-CaO_2_ nanoparticles exhibited a higher percentage of Ca^2+^ release than CaO_2_ nanoparticles under the same conditions. This could be attributed to the PAA modification effectively preventing the aggregation of CaO_2_ nanoparticles, making the PAA-CaO_2_ nanoparticles more solubility and allowing for a larger surface area in contact with the buffer [32].

Ca^2+^ can cause blood coagulation by catalyzing the conversion of prothrombin to thrombin and is influenced by the concentration of Ca^2+^ [36]. PAA-CaO_2_ nanoparticles release Ca^2+^ that may induce blood coagulation, based on the *in vitro* Ca^2+^ release experiment [25]. The blood clotting effect in the presence of CaO_2_, PAA-CaO_2_ nanoparticles and normal saline was investigated at the above pH values *in vitro* (Supplementary Fig. S6). For normal saline, no blood clotting was observed at all pH values. Nevertheless, PAA-CaO_2_ and CaO_2_ nanoparticles induced blood clotting at pH 6.5 with a blotting time of 1 and 18 min, respectively. The results showed that the PAA-CaO_2_ nanoparticles compared to CaO_2_ nanoparticles cause faster blood clotting. Similarly, with increased acidity, PAA-CaO_2_ nanoparticles evoked more rapid blood clotting at pH 6.5 compared to pH 7.4. Simultaneously, biocompatibility tests confirmed that PAA-CaO_2_ nanoparticles were blood compatible at physiological conditions (Supplementary Fig. S7). This unique property of pH-sensitively inducing blood coagulation endows PAA-CaO_2_ nanoparticles to be a potential material for adequate hemostasis in wound therapy [8], especially in acidic environments commonly found in pathological wounds.

### *In vitro* antibacterial activity

**Figure 3.**
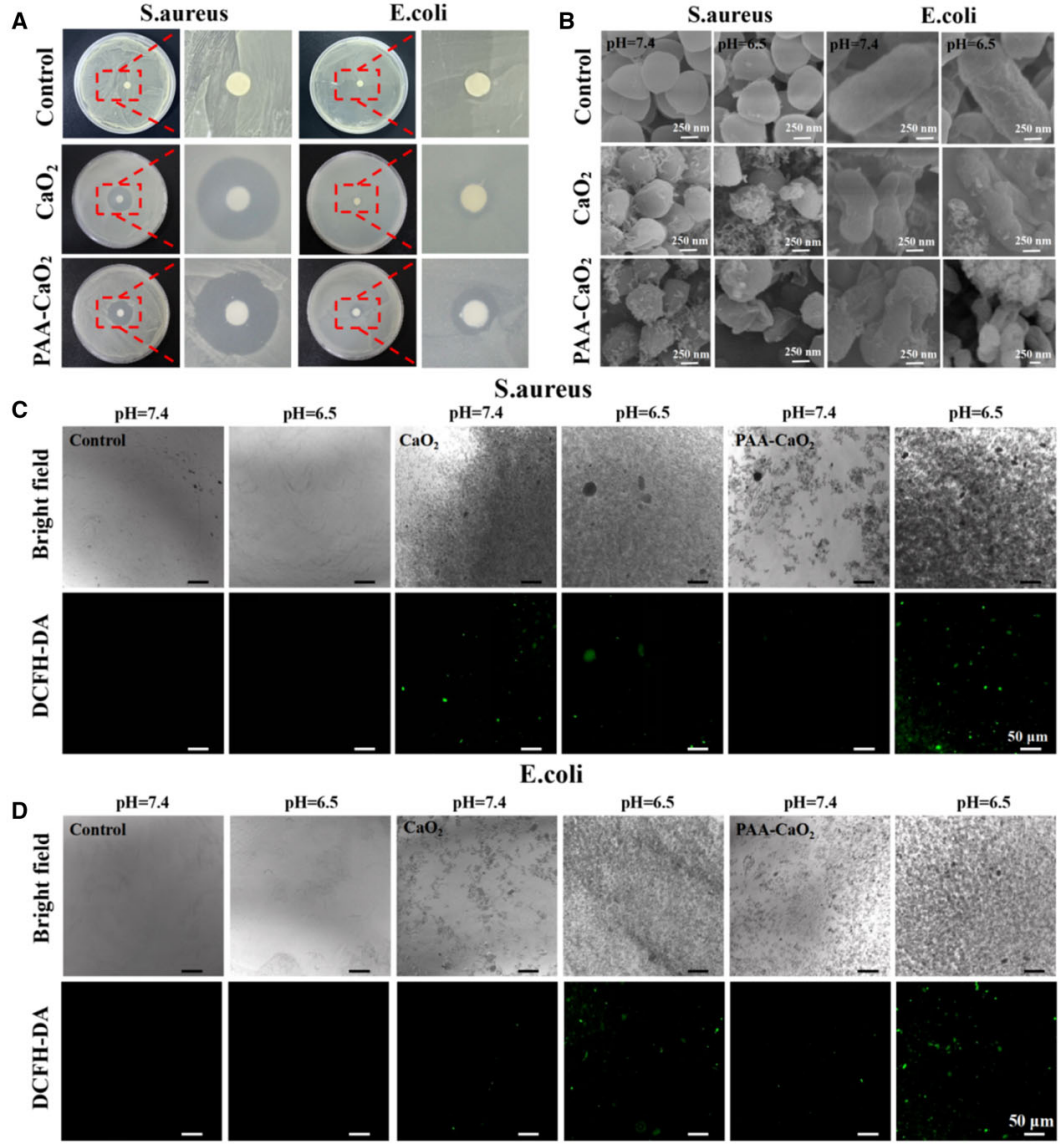
(**A**) The Antibacterial ring of *S.aureus* and *E.coli* LB agar plates after treatment with corresponding formulas. (**B**) SEM images of *S.aureus* and *E.coli* after treatment with corresponding formulas at different pH values. Bright-field and DCFH-DA fluorescence images of *S.aureus* (**C**) and *E.coli* (**D**) bacterial strains after being treated with corresponding formulas at different pH values, respectively.

Bacteria can invade the body from the skin when a wound occurs, likewise in other traumas or surgeries. Invading bacteria and other microorganisms into wounds is the leading cause of severe infections [37]. Antibacterial tests were performed with PAA-CaO_2_ nanoparticles to evaluate their antibacterial potential in two representative bacterial strain models (*E.coli* and *S.aureus*) coinciding with a previous study [19, 38]. The results are shown in Fig. 3A; PAA-CaO_2_ nanoparticles showed the largest area of antibacterial rings for *E.coli* and *S.aureus* bacterial strains. We further found that PAA-CaO_2_ nanoparticles showed higher antibacterial activity against Gram-positive bacteria (*S.aureus*) than Gram-negative bacteria (*E.coli*). In the Gram-negative bacterial group, the inhibition zone of PAA-CaO_2_ nanoparticles exhibited a moderate-size ring. In contrast, the inhibition zone of the PAA-CaO_2_ nanoparticles treatment group in Gram-positive bacteria was as high as 8.97 ± 0.26 cm^2^, significantly higher than the normal saline group (control). It can be considered that metal peroxide nanoparticles are more virulent against Gram-positive bacteria than Gram-negative bacteria [39]. We speculated that there are different structures between Gram-positive and Gram-negative bacteria. The mechanism of antibacterial activity of metal and metal peroxide nanoparticles involves multiple factors, including membrane damage, metal ions and ROS that affect bacteria metabolism. For example, Gram-positive bacteria have a thicker peptidoglycan layer than Gram-negative bacteria. Nonetheless, Gram-negative bacteria have relatively less permeable outer membranes surrounded by lipids and proteins, which may lead to higher barriers to the penetration of nanoparticles and metal ions into bacteria. In addition, the surface of Gram-positive bacteria is more negatively charged, which helps attract positive ions, such as Ca^2+^ [40–42]. The acid produced in the bacteria caused the release of highly toxic ROS from PAA-CaO_2_ nanoparticles, resulting in the massive death of bacteria in contact with the nanoparticles. Additionally, it was confirmed in subsequent WB experiments that PAA-CaO_2_ nanoparticles have an antibacterial effect by activating the MEK1/2/ERK1/2 pathway to stimulate neutrophil cells to adhere to endothelium. Meanwhile, *S.aureus* tends to be a common bacterium on the skin in the existing reports, making it the leading cause of general wound infection [43]. This phenomenon stimulated our interest in further analysis of PAA-CaO_2_ nanoparticles in *S.aureus*-infected wounds.

Simultaneously, the effects of PAA-CaO_2_ nanoparticles on bacterial morphology at different pH values were investigated. Not surprisingly, the bacterial membrane surfaces in the control group remained intact in acidic and neutral buffer solutions. However, when the bacteria were treated with PAA-CaO_2_ nanoparticles, the bacterial membrane was damaged more severely in an acidic solution than in a neutral solution (Fig. 3B). This is related to a large amount of ROS released by PAA-CaO_2_ nanoparticles under acid stimulation, oxidizing the bacterial membrane, resulting in the leakage of bacterial contents [44, 45]. Compared with the control group, PAA-CaO_2_ nanoparticles exhibited a more robust and stable antibacterial effect in the wound atmosphere.

We used DCFH-DA as a fluorescent probe to visualize ROS production in bacteria [46]. After incubation of two bacteria with PAA-CaO_2_ nanoparticles for 2 h, obvious green fluorescence was observed in acidic buffer, but almost no fluorescence was observed under neutral condition (Fig. 3C and D). This could mimic wound microenvironment [47] and protons produced by the bacteria existing in the wound resulting in certain ROS being produced. The results suggested that releasing toxic ROS is acid-dependent and applying in infected wound is beneficial. The reason is clear that the dissociation rate of PAA-CaO_2_ nanoparticles is abruptly accelerated in an acidic environment, depending on the protons concentration around the nanoparticles, compared to the slow hydrolysis process that relies on protons generated by water ionization under neutral conditions. The chemical reaction equation can be written as follows: CaO_2_ + 2H^+^ = Ca^2+^ + H_2_O_2_. Meanwhile, the findings revealed that peroxo groups were present in PAA-CaO_2_ nanoparticles, which aligns with the XPS results.

### *In vitro* cell experiments

**Figure 4.**
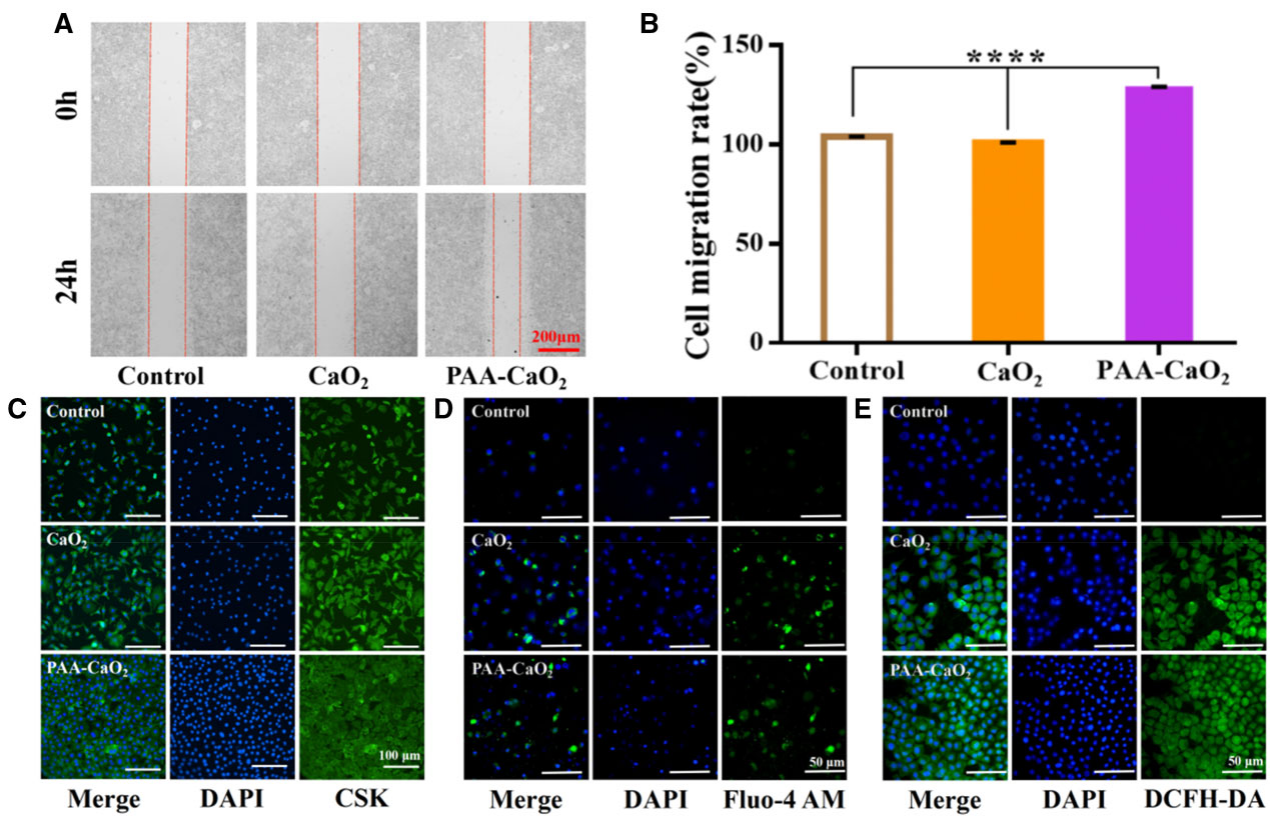
*In vitro* migration, proliferation assays and intracellular Ca^2+^ and ROS detection assays of L929 cells. (**A**) Migration assay and (**B**) corresponding statistical analysis by ImageJ (****P < 0.0001). (**C**) Fluorescence characterization of cell proliferation images. Schematic diagram of intracellular Ca^2+^ (**D**) detection and ROS (**E**) detection, respectively.

Given the antibacterial properties and controlled release effects of PAA-CaO_2_ nanoparticles, we further explored the impact of PAA-CaO_2_ nanoparticles on cell proliferation and migration. Fibroblasts proliferate to form contractile granulation tissues, which are crucial to wound healing [48]. Hence, scratch assays were performed on the mouse epithelioid fibroblasts L929 cells to investigate the effects of PAA-CaO_2_ nanoparticles on migration (Fig. 4A). Simultaneously, the migration results were quantified in Fig. 4B. The results revealed that PAA-CaO_2_ nanoparticle-treated group had the highest ability to accelerate cell migration.

Furthermore, the CCK-8 assay demonstrated the ability of PAA-CaO_2_ nanoparticles to promote cell proliferation and was more robust than CaO_2_ nanoparticles at an optimum concentration (Supplementary Fig. S8). Next, fluorescent staining was performed on the L929 cell line and verified that PAA-CaO_2_ nanoparticles have a salient promoting effect on cell proliferation (Fig. 4C). This phenomenon is due to the released Ca^2+^ and ROS in PAA-CaO_2_ nanoparticles described previously. Ca^2+^ and ROS can modulate fibroblast proliferation/migration, enhancing angiogenesis [8, 11]. To confirm that PAA-CaO_2_ nanoparticles did produce Ca^2+^ and ROS that could promote cell proliferation and migration at the cellular level. It was examined by fluorescent probes and monitored by CLSM. The control group exhibited weak green luminescence, while the group cultivated with PAA-CaO_2_ nanoparticles exhibited strong intracellular luminescence, indicating the release of the exogenous free Ca^2+^ (Fig. 4D) and ROS (Fig. 4E) from PAA-CaO_2_ nanoparticles. The experimental cell results collectively provided a basis for *in vivo* wound healing. It is reasonable to expect that Ca^2+^ and ROS would play regulatory roles, promoting wound healing.

### Accelerated wound healing in full-thickness skin defect rats

**Figure 5.**
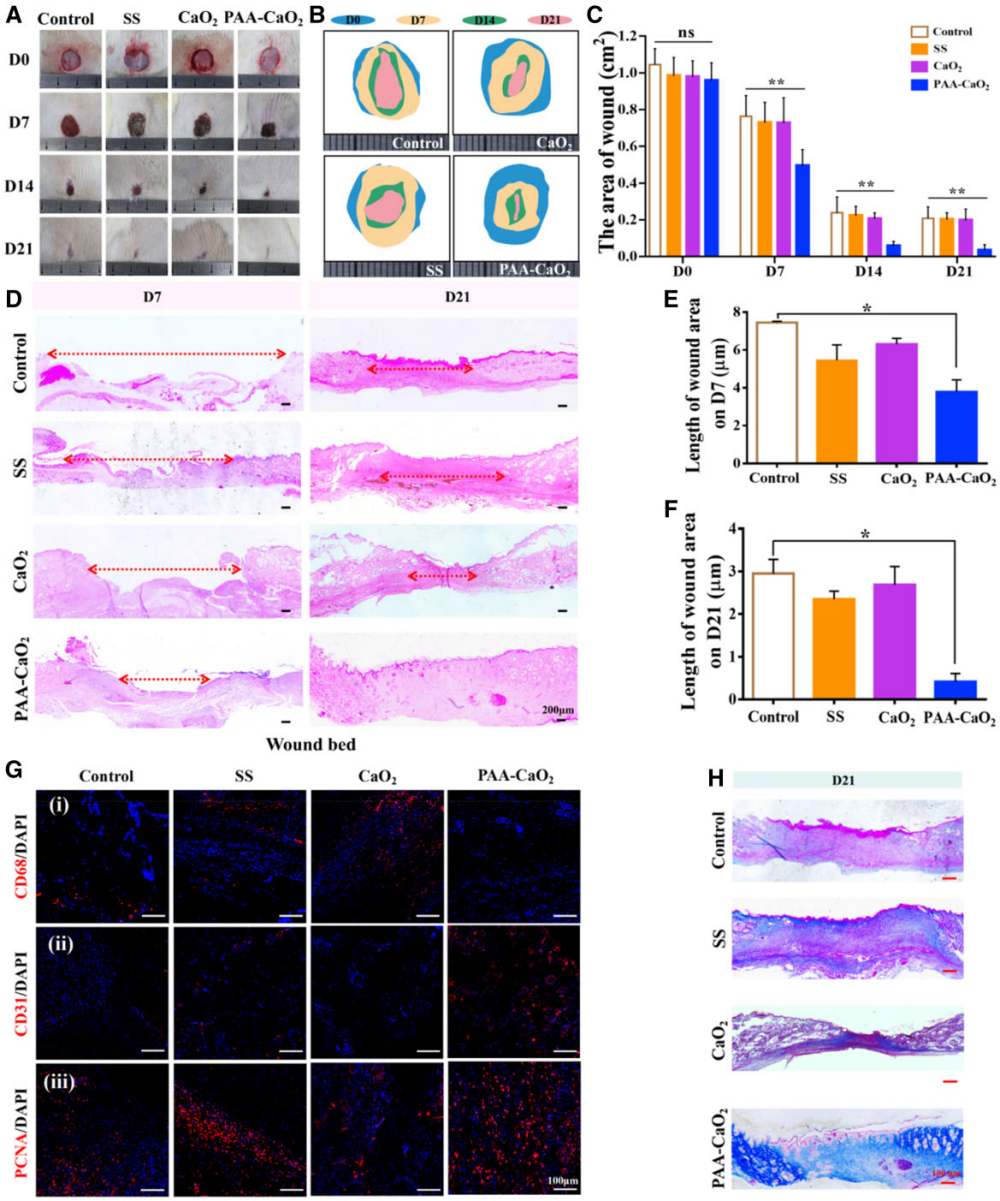
(**A**) Overview of the size change of wound status at 0, 7, 14 and 21 days after differently treating with normal saline (control), SS, CaO_2_ and PAA-CaO_2_ nanoparticles, respectively. Relative area diagram (**B**) and statistics (**C**) of the wound. (**D**) H&E staining of the wound bed at 7 and 21 days. Corresponding statistics of wound bed length in H&E staining at 7 (**E**) and 21 (**F**) days. (**G**) IF images of the regenerated skin tissues labeled with (i) CD68 on Day 3, and (ii) CD31, (iii) PCNA on Day 7 after treatment with different formulas. (**H**) The collagen deposition on Day 21 (ns: non-significant, *P < 0.05, **P < 0.01).

We studied the therapeutic effects of PAA-CaO_2_ nanoparticles for wound treatment in a full-thickness skin defect rat model. Besides, we used SS as a positive control group for comparison with marketed skin repair drugs [19, 49]. As shown in Fig. 5A and B, the wound region in all groups became smaller while the wounds treated with PAA-CaO_2_ nanoparticles healed. On Day 7, the wound area was significantly reduced as PAA-CaO_2_ nanoparticles inhibited the inflammatory response in the early stage of healing and regulated the initial effective wound healing. On the 14th day, the defected area of the PAA-CaO_2_ nanoparticles group decreased more than the other groups, indicating that PAA-CaO_2_ nanoparticles still had superior repair performance in the middle stage of wound repair. On the 21st day, the PAA-CaO_2_ nanoparticles group exhibited wound healing. At the same time, visible scabs remained in the other groups. The wound healing rate histogram statistically displayed that the wound repair effects of the PAA-CaO_2_ nanoparticles group were the best (Fig. 5C). Meanwhile, we have taken different samples from Day 7 to Day 21 for H&E staining. There were notably larger wound beds in the control, SS and CaO_2_ groups than in PAA-CaO_2_ nanoparticles group on Day 7 and Day 21 (Fig. 5D). The corresponding quantified statistics of wound beds at 7 (Fig. 5E) and 21 days (Fig. 5F) claim that PAA-CaO_2_ nanoparticles possessed satisfactory wound repair performance *in vivo*.

### Reduced inflammatory reaction

Many biological factors, such as inflammation, angiogenesis and collagen deposition, can influence wound healing [3]. To investigate whether PAA-CaO_2_ nanoparticles induced wound healing by impacting inflammation, angiogenesis or collagen deposition. IF and Masson’s trichrome staining were conducted in succession. On Day 3 after injury, the wound area revealed an intense inflammatory response. Excessive inflammation is an additional component that might impede wound healing. Nevertheless, an effective immune response to local wounds during the early phases of recovery promotes the regeneration of the vascular network and the elimination of metabolic byproducts of the lesion [50]. We investigated the state of the inflammatory cell’s density in the early phases using the CD68 IF labeling to identify M1 macrophages in a pro-inflammatory state detrimental to wound healing, characterized by the production of several pro-inflammatory mediators [51]. We demonstrated the PAA-CaO_2_ nanoparticles group exhibiting the lowest density of adverse immune cells, indicating that the nanoparticles could alleviate the immunoreaction to a certain extent (Fig. 5G, i). It may be attributed to the PAA-CaO_2_ nanoparticles’ initial bacterial elimination ability to mediate it, thereby accelerating wound closure.

### Promoted angiogenesis and cell proliferation

Angiogenesis is essential as it requires the nutrients to be transported in the blood to the sites of new tissue creation for wound healing [48]. CD31 IF staining of vasculature in a histologic section was used to characterize the new vessels in wounds (Fig. 5G, ii). The total number of microvessels in the PAA-CaO_2_ nanoparticles group indicated a considerable increase compared to the other groups, enhancing angiogenesis. In addition, the formation of new structures is linked to the proliferation of diverse cells within the wound. PCNA is a crucial element in embodying the proliferative capacity of cells *in vivo*. By labeling cells with an anti-PCNA antibody, we analyzed cell proliferation vigor. The wounds treated with PAA-CaO_2_ nanoparticles exhibited the greatest strength of fluorescent highlights (Fig. 5G, iii). These results revealed that PAA-CaO_2_ nanoparticles showed anti-inflammatory activity, increased cell proliferation and angiogenesis *in vivo* [52–55].

### Enhanced collagen deposition

Collagen fibers trigger extracellular matrix (ECM) remodeling. Several steps in the healing process involved the deposition of collagen [56]. In this study, we examined that PAA-CaO_2_ nanoparticles affect collagen deposition by Masson's trichrome staining. The collagen fibers (stained with aquamarine blue) in the PAA-CaO_2_ nanoparticles group were more in number and more likely to form organized networks. The other groups displayed disorganized collagen structure and visible scars (Fig. 5H). We explained that excessive inflammatory reaction would enhance the overexpression of matrix metalloproteinases, destroying the ECM component and inhibiting collagen deposition [21]. While PAA-CaO_2_ nanoparticles might reduce inflammation and subsequently improve collagen deposition.

### Wound-induced transcriptomic analysis upon PAA-CaO_2_ nanoparticles treatment

**Figure 6.**
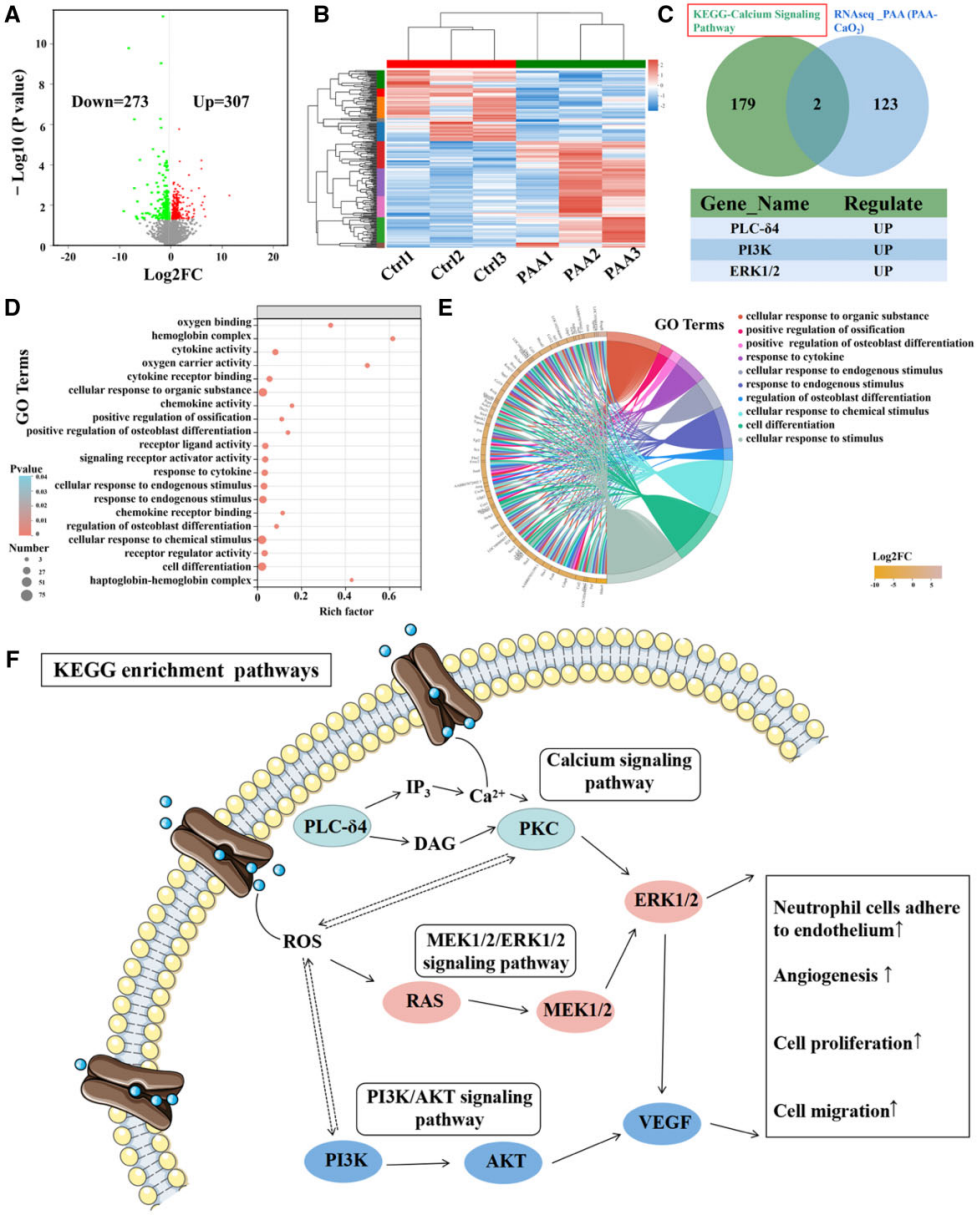
Transcriptomic analysis of rats treated with normal saline (control, ctrl) and PAA-CaO_2_ nanoparticles (PAA). (**A**) Volcano plot of RNA-seq data of wound in the two groups calculated by DESeq2 (*n* = 3 per group). (**B**) Heat map of DEGs between the two groups (*n* = 3 per group). (**C**) Venn analysis to screen hub genes involved in enrichment analysis and up-regulated DEGs. (**D**) GO bubble plot and (**E**) GO chord plot of GO enrichment analysis, displaying the relationship between genes and GO terms. (**F**) The KEGG enrichment pathway diagram of PAA-CaO_2_ nanoparticles enhances calcium, PI3K/AKT and MEK1/2/ERK1/2 signals to regulate wound healing processes. PLC-δ4, phospholipase C Delta 4; DAG, diacylglycerol; IP_3_, inositol 1, 4, 5-trisphosphate; PKC, protein kinase C; PI3K, phosphoinositide 3-kinase; AKT, protein kinase B; MEK1/2, mitogen-activated protein kinase kinase (MAPKK); ERK1/2, extracellular signal-regulated kinase; VEGF, vascular endothelial growth factor.

To better understand the antibacterial and wound-healing mechanisms behind the impact of PAA-CaO_2_ nanoparticles, we conducted a broad transcriptome analysis of a wound treated with PAA-CaO_2_ nanoparticles as an initial step. Our analysis identified 580 differentially expressed genes in the volcano analysis following treatment with PAA-CaO_2_ nanoparticles. Among these genes, 307 were upregulated, while 273 were downregulated (Fig. 6A and B). To gain insights into the impact of PAA-CaO_2_ nanoparticles on cell proliferation, we performed a GO-Chord analysis on the enriched items related to this effect, which offered a comprehensive understanding of the functional roles and interactions of the enriched genes in these specific biological processes. The enriched genes identified in the GO-Chord and GO-Bubble plots were assigned to several categories, including ‘cell differentiation’, ‘response to cytokine’, ‘cellular response to endogenous stimulus’ and ‘oxygen carrier activity’ (Fig. 6D and E). These findings provide valuable insights into the molecular changes induced by treatment with PAA-CaO_2_ nanoparticles, particularly concerning their effects on wound healing.

Consistently, we performed KEGG enrichment analysis to explore the initial driver of the wound healing effect of PAA-CaO_2_ nanoparticles treatment [57]. It found that these differently expressed genes mainly enriched in the ‘calcium signaling pathway’, ‘PI3K/AKT signaling pathway’ and ‘MEK1/2/ERK1/2 signaling pathway’, including PLC-δ4, PI3K, AKT, MEK1/2 and ERK1/2. Notably, the expression level of wound healing-promoting genes such as PLC-δ4 (an early signal in the calcium signaling pathway) [58], PI3K and ERK1/2 were found to be upregulated (Fig. 6C). These findings shed light on the potential mechanism through which PAA-CaO_2_ nanoparticles systemically induce wound repair.

### Calcium, PI3K/AKT and MEK1/2/ERK1/2 signaling pathways driven PAA-CaO_2_ nanoparticles-mediated wound healing

After analyzing the transcriptome and protein–protein interaction (PPI) network analysis of different formulas treated wounds, we have identified the calcium, PI3K/AKT and MEK1/2/ERK1/2 signaling pathways as crucial events in PAA-CaO_2_ nanoparticle-mediated wound healing. The PI3K/AKT pathway is vital in numerous cellular functions, such as signal transmission for cell adhesion, growth, proliferation, migration and angiogenesis [59]. While the activation of intracellular signaling pathways, like mitogen-activated protein kinase (MAPK) signaling, further promotes wound healing. MAPK kinase (MEK1/2), a kinase enzyme, phosphorylates MAPK, leading to the activation of MEK1/2 signaling. The MEK1/2/ERK1/2 signaling pathway is the most common one of the MAPK signaling pathways. Recently reported that catalpol promotes angiogenesis via crosstalk of the MEK1/2/ERK1/2 pathway [60]. Moreover, this signaling pathway also drives cell proliferation/migration of fibroblasts/keratinocytes and differentiation of fibroblasts, which are crucial events in wound healing [61].

Our concept involved the release of Ca^2+^ through the cytoplasmic mechanism, which we call ‘External Calcium Triggering’. This activates the calcium signaling pathway. PPI network analysis revealed that PAA-CaO_2_ nanoparticles activate the calcium signaling pathway through PLC-δ4. This pathway relies on PLC-δ4 to hydrolyze phosphatidylinositol 4,5-bisphosphate (PIP_2_) to generate two second messenger DAG and IP_3_. DAG mediates the activation of PKC, which could mediate multiple downstream cellular events, including transcription, proliferation and differentiation [62]. On the other hand, IP_3_ releases Ca^2+^ from intracellular stores, leading to Ca^2+^-dependent MEK1/2 signaling [63] and extracellular P-selectin expression, which endows PAA-CaO_2_ nanoparticles with antibacterial ability by enhancing neutrophil cells to adhere to the endothelium [64], subsequently neutrophil cells traveling to the source of infection. At the same time, the H_2_O_2_ (ROS) released from PAA-CaO_2_ nanoparticles can stimulate the MEK1/2 pathway to activate ERK1/2 [65]. In addition, an increase in vascular endothelial cells ROS also caused a release of stored Ca^2+^ from the endoplasmic reticulum into the cytosol. Consequently, Ca^2+^ and ROS can activate numerous molecules involved in wound healing.

**Figure 7.**
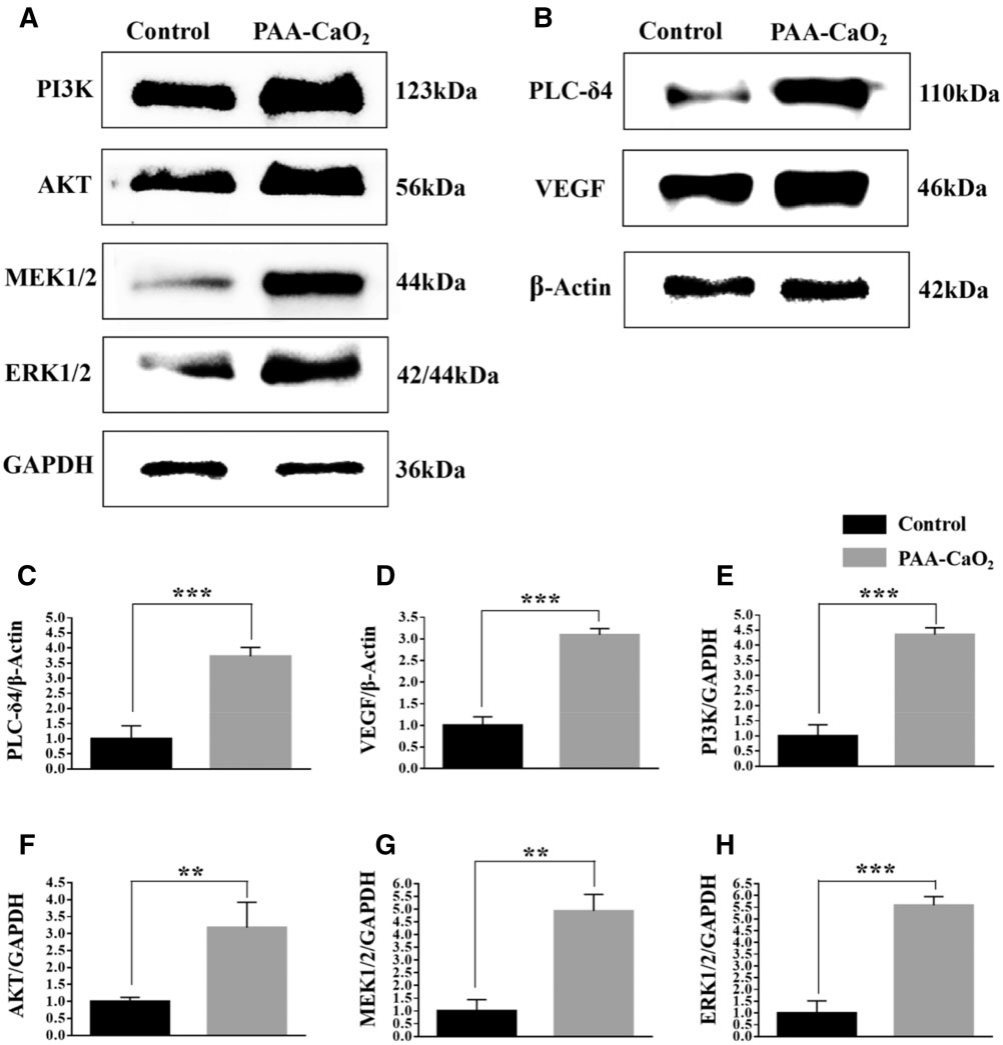
The protein expression level of the control and PAA-CaO_2_ nanoparticles treatment groups on Day 7. The PI3K, AKT, MEK1/2, ERK1/2 (**A**) and the PLC-δ4, VEGF (**B**) expression of the wound after incubation with different formulas. Quantification of PLC-δ4 (**C**), VEGF (**D**), PI3K (**E**), AKT (**F**), MEK1/2 (**G**) and ERK1/2 (**H**) expression level by ImageJ (**P < 0.01, ***P < 0.001).

WB was used to confirm the pathways of PAA-CaO_2_ nanoparticles-activated. In the PAA-CaO_2_ nanoparticles treated group, the expression of PI3K, AKT (Fig. 7A, E and F), MERK1/2, ERK1/2 (Fig. 7A, G and H) and PLC-δ4 (Fig. 7B and C) were markedly increased compared to the control group. These results suggested that the PI3K/AKT, MEK1/2/ERK1/2 and calcium signaling pathways were activated. Furthermore, we have proved that the critical molecule on angiogenesis-VEGF expression was significantly increased by PAA-CaO_2_ nanoparticles (Fig. 7B and D).

Overall, the present study demonstrated that PAA-CaO_2_ nanoparticles can induce angiogenesis, exert cell proliferation, migration subsequently accelerate wound healing *in vivo* by increasing the expression of PLC-δ4, PI3K, AKT, MEK1/2, ERK1/2 and VEGF. Moreover, activating the MEK1/2/ERK1/2 pathway to stimulate neutrophil cells to adhere to the endothelium enhanced the antibacterial ability of PAA-CaO2 nanoparticles. These biological processes were mediated by calcium, MEK1/2/ERK1/2 and PI3K/AKT signaling pathways together.

### Application in *S.aureus*-infected wound healing

We further evaluated the practical applicability of PAA-CaO_2_ nanoparticles in treating common clinical bacterial (*S.aureus*)-infected wounds due to its favorable properties. Due to the severity and possible delays in the recovery of infected wounds, we increased the frequency of dressing changes in each experimental group to accelerate bacterially infected wounds healing effectively. It is more suitable for spraying reagents’ applicability to infected wounds in clinical practice.

**Figure 8.**
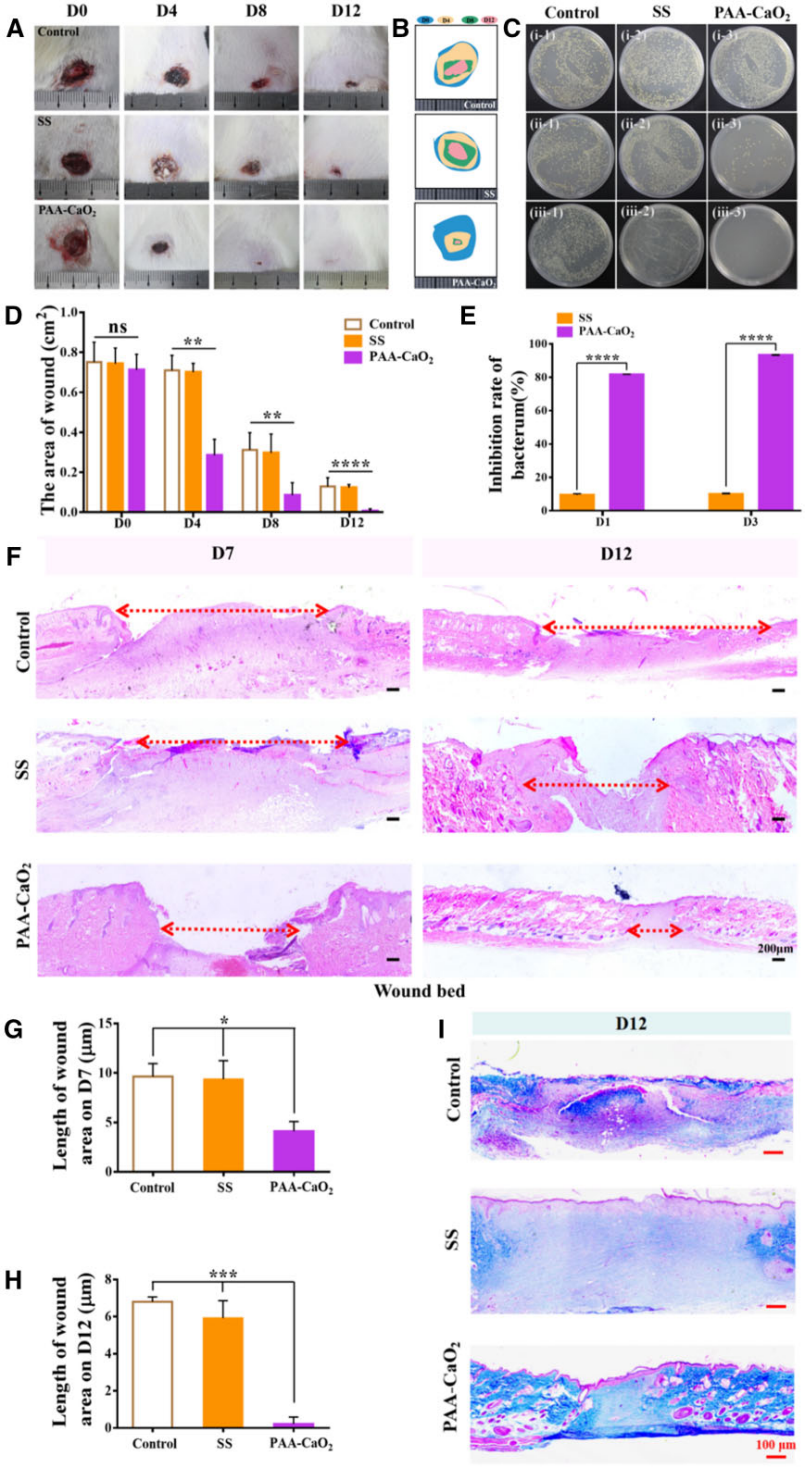
(**A**) Overview of the size change of *S.aureus*-infected wound status at 0, 4, 8 and 12 days after treating with normal saline (control), SS and PAA-CaO2 nanoparticles differently. Relative area diagram (**B**) and statistics (**D**) of the wound. (**C**) Infected wounds exudate bacterial colony culture coated plate after being treated with different formulas to post *S.aureus*-inhibition rate: (i) Day 0 (12 h after infection); (ii) Day 1; (iii) Day 3. (**E**) Quantitative analysis of the exudate bacterial colony in the wound by ImageJ. (**F**) H&E staining of the wound bed at 7 and 12 days. Corresponding statistics of wound bed length in H&E staining at 7 (**G**) and 12 (**H**) days. (**I**) The collagen deposition on Day 12 (ns: non-significant, *P < 0.05, **P < 0.01, ***P < 0.001, ****P < 0.0001).

In this experiment, the *S.aureus*-infected rats in different groups were severally treated with normal saline, SS and PAA-CaO_2_ nanoparticles. The wounds were observed and photographed on schedule. As shown, edema and inflammation were observed in the wounds on the back of all rats after the *S.aureus* infection (Day 0). After 4 days of treatment, compared to the control and SS groups, PAA-CaO_2_ nanoparticles-treated wound exuded relatively less, no apparent abscess appeared, and the wound area was much smaller. After 12 days of treatment, we observed that the wounds in the PAA-CaO_2_ nanoparticles group were almost healed, while the control and SS groups had distinct wounds (Fig. 8A and B). There was a significant difference between PAA-CaO_2_ nanoparticles and the other two treatment groups (Fig. 8D).

Simultaneously, to quantitatively evaluate the bactericidal effects of PAA-CaO_2_ nanoparticles, we collected abscess exudate in the first 3 days to quantify the number of bacteria in the wound area (Fig. 8C). From the grown colony, we found that PAA-CaO_2_ nanoparticles exhibited the most effective bacterial elimination ability (Fig. 8E). Infected wounds treated with PAA-CaO_2_ nanoparticles had conspicuous power in killing bacteria on the second day, with almost no colony growth. The therapeutic effects of the infected wounds *in vivo* further confirmed that PAA-CaO_2_ nanoparticles have higher feasibility and practical applications in the clinical therapy of infected wounds. It also demonstrated that Ca^2+^ and ROS released by PAA-CaO_2_ nanoparticles had higher antibacterial activity than silver ions in SS.

Similarly, we have taken different treatment groups samples for H&E staining. In the control and SS groups, there were large wound beds than the PAA-CaO_2_ nanoparticles group (Fig. 8F) with corresponding statistics analysis on Day 7 (Fig. 8G) and 12 (Fig. 8H). Simultaneously, more collagen fibers, organized collagen structure and the smallest scar tissues were discovered in the PAA-CaO_2_ nanoparticles group (Fig. 8I). Suggesting PAA-CaO_2_ nanoparticles possess satisfactory *S.aureus*-infected wound repair performance *in vivo*.

## Conclusions

This work developed a novel nanocarrier, PAA-CaO_2_ nanoparticles, which are biocompatible, pH-responsive, and have an appropriate size for drug administration. Moreover, they are economically available in large quantities through a one-pot method. PAA-CaO_2_ nanoparticles could release Ca^2+^ and H_2_O_2_ (ROS), and both two can mediate calcium, PI3K/AKT and MEK1/2/ERK1/2 signaling pathways together to antibacterial, reinforce angiogenesis, cell proliferation/migration in the wound microenvironment. Experiments were conducted on general and infected wounds in SD rats to evaluate the healing effects of PAA-CaO_2_ nanoparticles *in vivo*. By the 21st postoperative day, the general wound in injured rats nearly recovered due to the PAA-CaO_2_ nanoparticles administration. PAA-CaO_2_ nanoparticles owed remarkable antibacterial activity *in vivo*, mainly when applied in *S.aureus*-infected rats. The results showed nearly no bacterial growth in the PAA-CaO_2_ nanoparticles group 2 days after treatment. Besides, compared with other reported materials involving the exploration of antibacterial and wound healing performance, PAA-CaO_2_ nanoparticles are relatively sufficient in these sections (Supplementary Table S1). Therefore, we designed PAA-CaO_2_ nanoparticles by fully exploiting Ca^2+^’s distinctive wound-healing mediated mechanism, showing that calcium signaling is as crucial as ROS to wound repair. Because of its pH sensitivity, PAA-CaO_2_ nanoparticles progressively break into Ca^2+^ and H_2_O_2_ in the wound area, antibacterial and accelerating tissue repair. Hence, PAA-CaO_2_ nanoparticles will provide promising employment for clinical chronic and infected wound therapy.

## Supplementary Material

rbad071_Supplementary_Data
