# Supplementary material for: Sprayed PAA-CaO2 nanoparticles combined with calcium ions and reactive oxygen species for antibacterial and wound healing
Source: Regen Biomater. 2023 Aug 21;10:rbad071. doi: 10.1093/rb/rbad071 (PMC10503269; doi:10.1093/rb/rbad071)
Supplement: rbad071_Supplementary_Data [file rbad071_supplementary_data.zip › The FINAL versions of Supplementary materials (RB-2023-079-R2).pdf]

Supplementary materials

Sprayed PAA-CaO_2 nanoparticles combined with calcium ions and reactive oxygen species for antibacterial and wound healing

Hong Yu[a,1], Jiale Sun[a,1], Kepeng She[a,1], Mingqi Lv[a,1], Yiqiao Zhang[a], Yawen Xiao[a], Yangkun Liu[a], Changhao Han[a], Xinyue Xu[a], Shuqing Yang[d,E,*], Guixue Wang[b,c,*], Guangchao Zang ,[a,b,c,*]

[a] Tissue and Cell Biology, Lab Teaching & Management Center, Chongqing Medical University, Chongqing, 400016, China

[b] Key Laboratory for Biorheological Science and Technology of Ministry of Education, State and Local Joint Engineering Laboratory for Vascular Implants, Bioengineering College of Chongqing University, Chongqing, 400030, China

[c] Jinfeng Laboratory, Chongqing, 401329, China

[d] Chongqing University Central Hospital, Chongqing Emergency Medical Center, Chongqing, 400030, China

*Correspondence should be addressed to Guixue Wang; wanggx@cqu.edu.cn, Guangchao Zang; zangguangchao@cqmu.edu.cn, and Shuqing Yang; yangshuqing72@163.com.

[1] These authors contributed equally to this work.

MATERIALS AND REAGENTS

25% $NH_3 \cdot H_2O$ and 30% H_2O_2 were purchased from ChuanDong Chemical (Chongqing, China). Polyacrylic acid and $CaCl_2$ were purchased from Aladdin (Shanghai, China). Dialysis bag was the product of Union Carbide (Danbury, Connecticut, USA). NaCl, Fluorescein Isothiocyanate (FITC), and FITC-conjugated Phalloidin were bought from Sangon Biotech (Shanghai, China). *Staphylococcus aureus* and *Escherichia coli* were supplied by the Pathogenic Microbiology Teaching and Research Laboratory (Chongqing Medical University, Chongqing, China).

Bovine serum albumin (BSA) was purchased from Solarbio Life Sciences (Beijing, China). Agar, tryptone, yeast extract, and DMEM medium were purchased from Thermo Fisher Scientific-CN (USA). Fetal Bovine Serum (FBS) was purchased from Biological Industries (Shanghai, China). Penicillin-Streptomycin (P/S) was purchased from HyClone (USA). The L929 cell line (Mouse Fibroblasts Cells, NCTC clone 929) was purchased from Icellbioscience (Shanghai, China). Paraformaldehyde was purchased from Leagene (Beijing, China). Antibodies against β-Actin, GAPDH, and secondary HRP labeled goat anti-rabbit IgG (H+L) antibody. Primary antibodies CD31, CD68, VEGF, and PCNA were purchased from Proteintech (Wuhan, China). Primary antibodies PLC-δ4, PI3K, AKT, MEK1/2, and ERK1/2 were purchased from Beyotime Biotechnology (Shanghai, China). DAB Chromogenic Kit was purchased from Solely Biological (Beijing, China). 2′,7′-Dichlorofluorescin diacetate (DCFH-DA) and the Bicinchoninic acid assay reagent (BCA) kit was obtained from Beyotime Biotechnology (Shanghai, China). Sulfadiazine silver cream is the product of HengJian (Guangdong, China). Male adult SD rats (Clean level) were supplied by the Experimental Animal Center of Chongqing Medical University. All other materials in this study were at least reagent grade and purchased from commercial sources and used as received. All chemicals in this study were analytical grade and all the regents were dissolved in deionized water. The water purification machine is the product of Hitech Instrument Co., Ltd. (Shanghai, China).

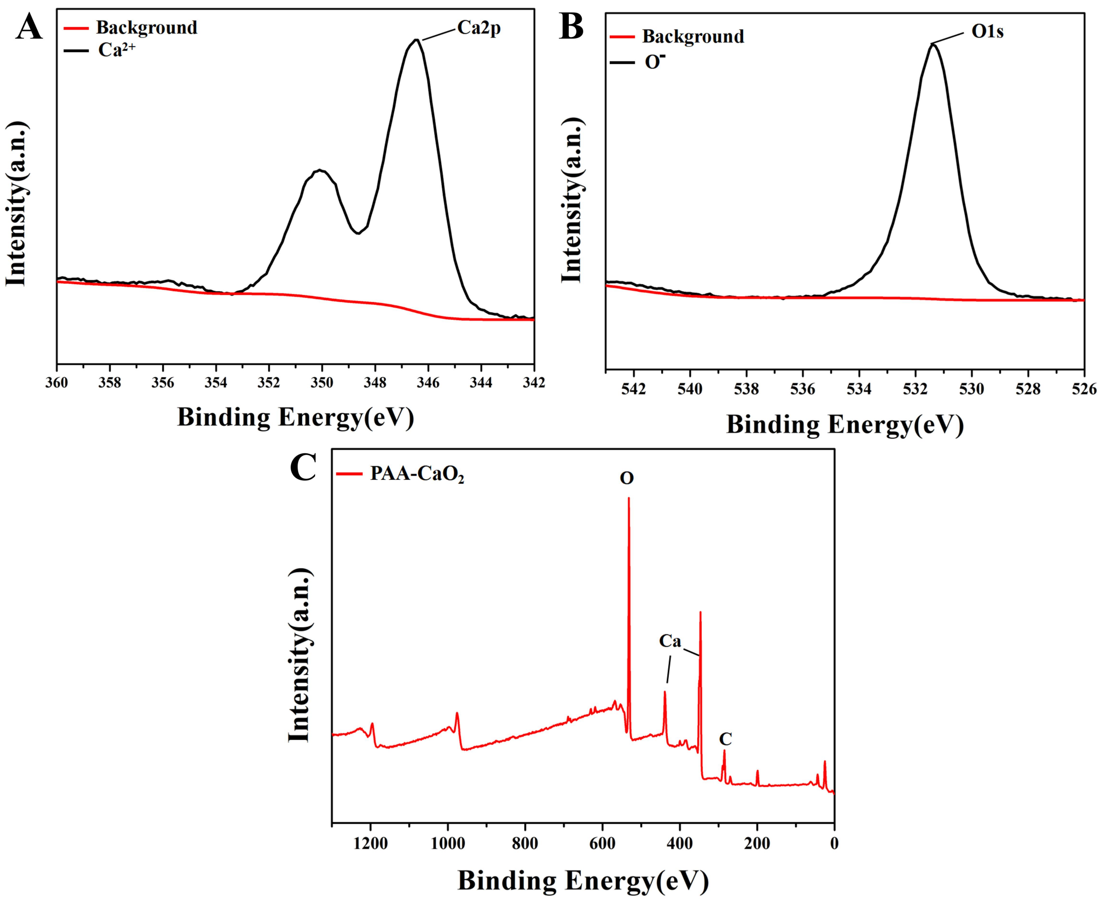

Fig.S1 (A) High resolution Ca 2p spectrum, (B) high resolution O 1s spectrum and (C) full spectrum XPS survey of PAA-CaO_2 nanoparticles.

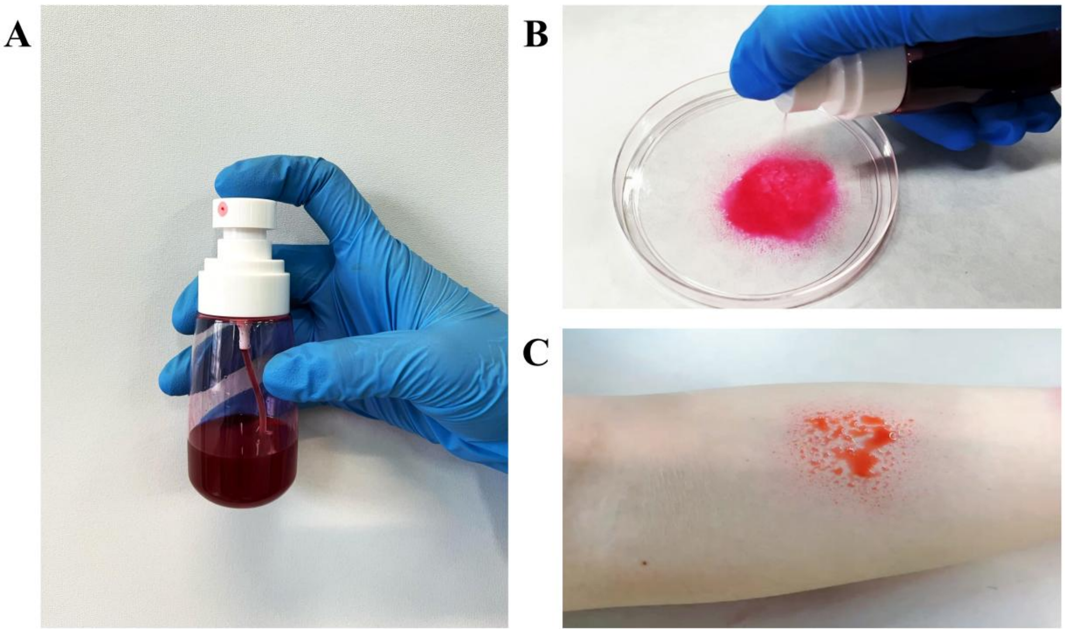

Fig.S2 Illustration of the sprayability of PAA-CaO_2 nanoparticles. (A) PAA-CaO_2 nanoparticles were mixed with a red food dye and bottled into a commercial plastic

spray bottle at room temperature. (B) PAA-CaO_2 nanoparticles could be easily sprayed onto a plastic disk and (C) human skin at room temperature.

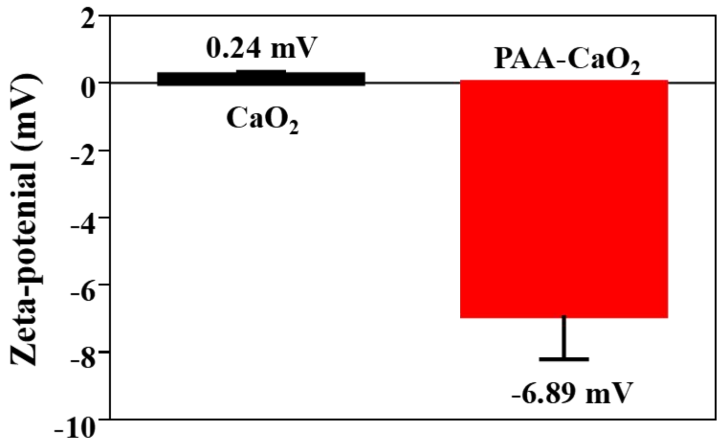

Fig.S3 Zeta potential of CaO_2 and PAA-CaO_2 nanoparticles.

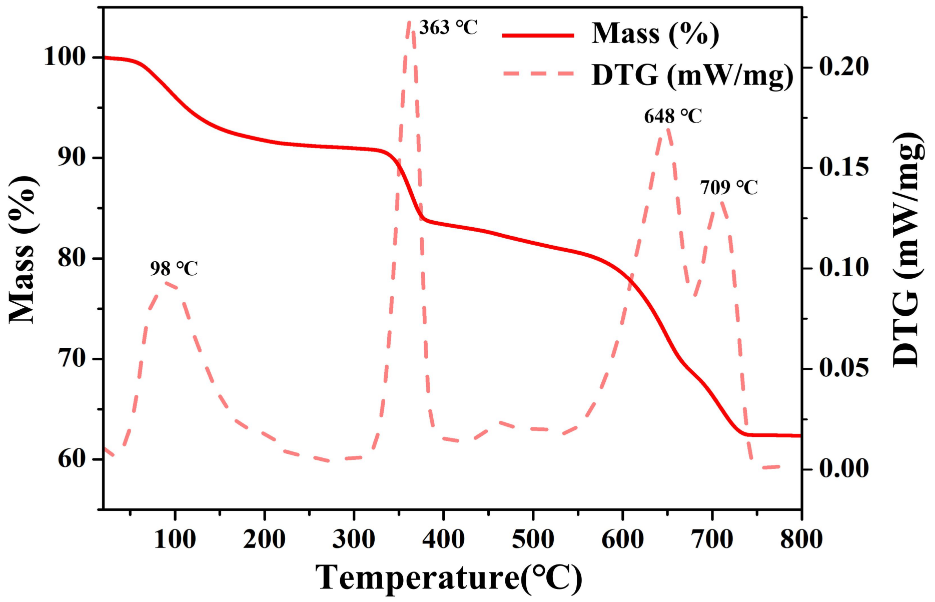

Fig.S4 TGA of PAA-CaO_2 nanoparticles.

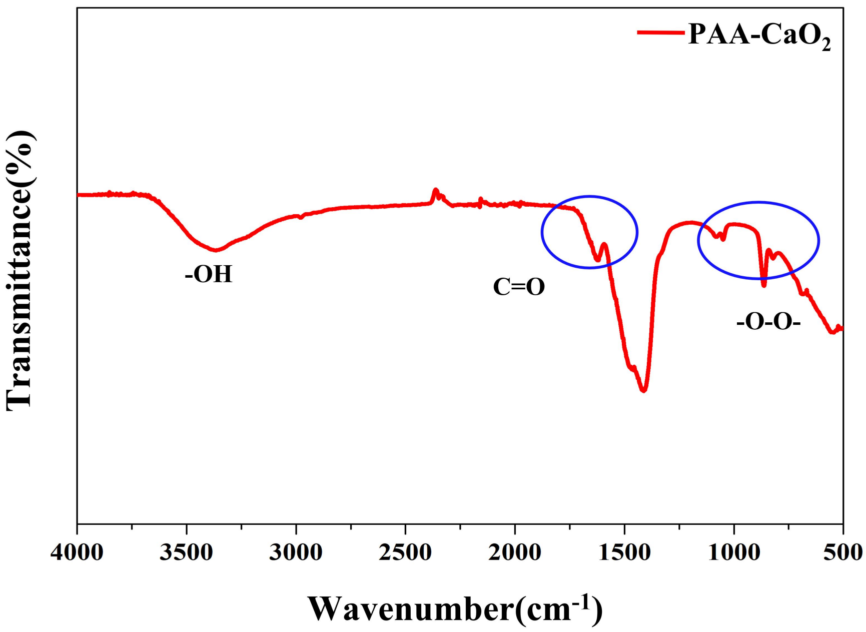

Fig.S5 FTIR spectra of PAA-CaO_2 nanoparticles.

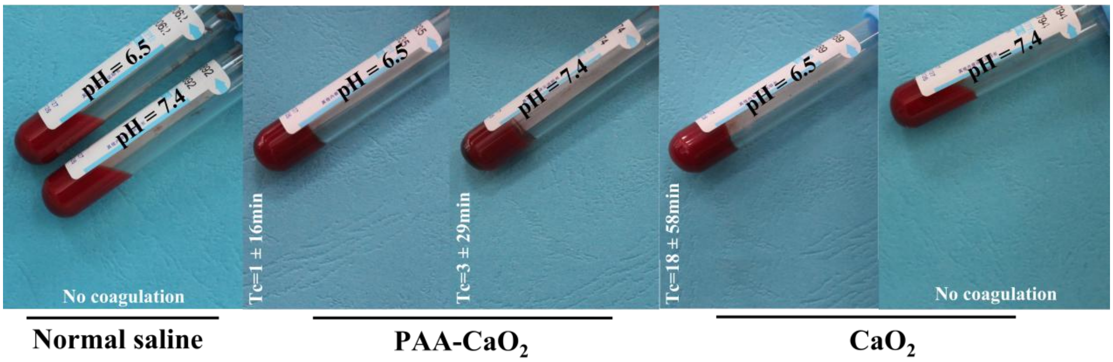

Fig.S6 Blood coagulation effect of normal saline, CaO_2, and PAA-CaO_2 nanoparticles suspension at different pH values in blood clotting test.

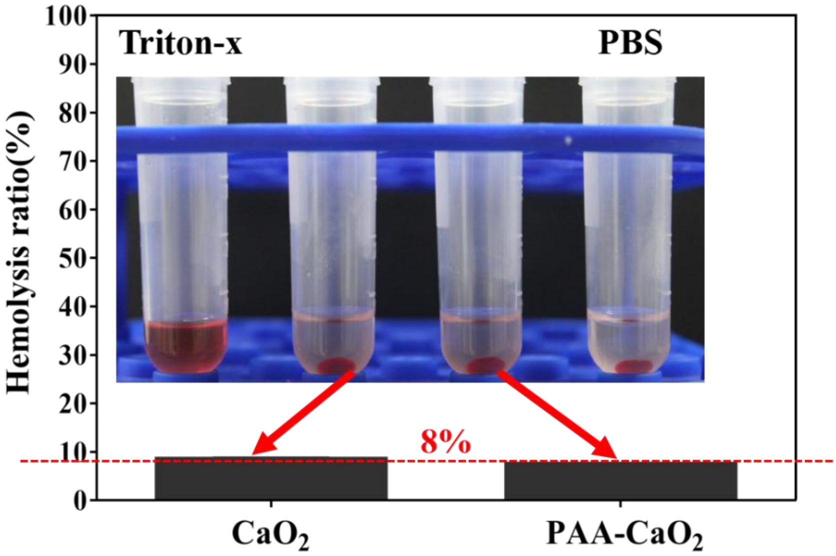

Fig.S7 Biocompatibility test of CaO_2 and PAA-CaO_2 nanoparticles. Triton-X treatment as the positive control group, and PBS treatment as the negative control group.

CCK-8 assay

A Ca^{2+} concentration of PAA-CaO_2 nanoparticles is 1 mg/ml showed the best ability to promote cell proliferation.

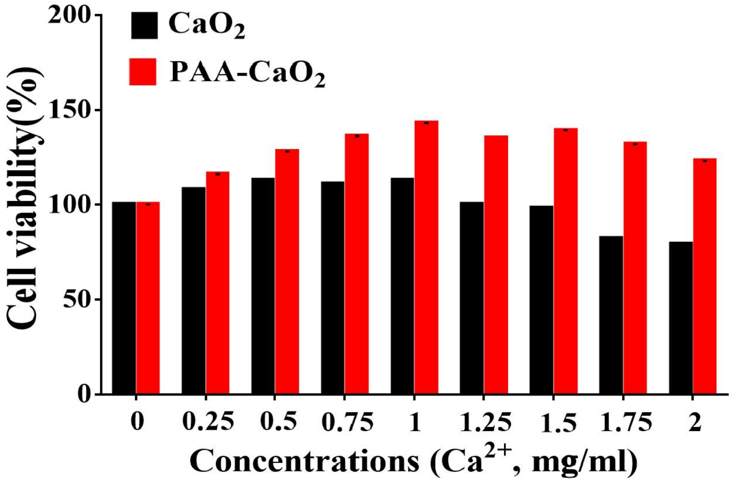

Fig.S8 CCK-8 assay. Cell viability of L929 treated with CaO_2 and PAA-CaO_2 nanoparticles at different concentrations.

Pilot toxicity study

Considering that PAA-CaO_2 nanoparticles have ROS-releasing capacity, which could have potential toxicity to organisms, the toxicity of PAA-CaO_2 nanoparticles to major

organs in rats was examined by H&E staining. As shown in Fig. S9, PAA-CaO_2 nanoparticles exhibited no significant histological alterations or toxicity during treatment. Indicating it is biocompatible and can be used *in vivo* for wound therapy.

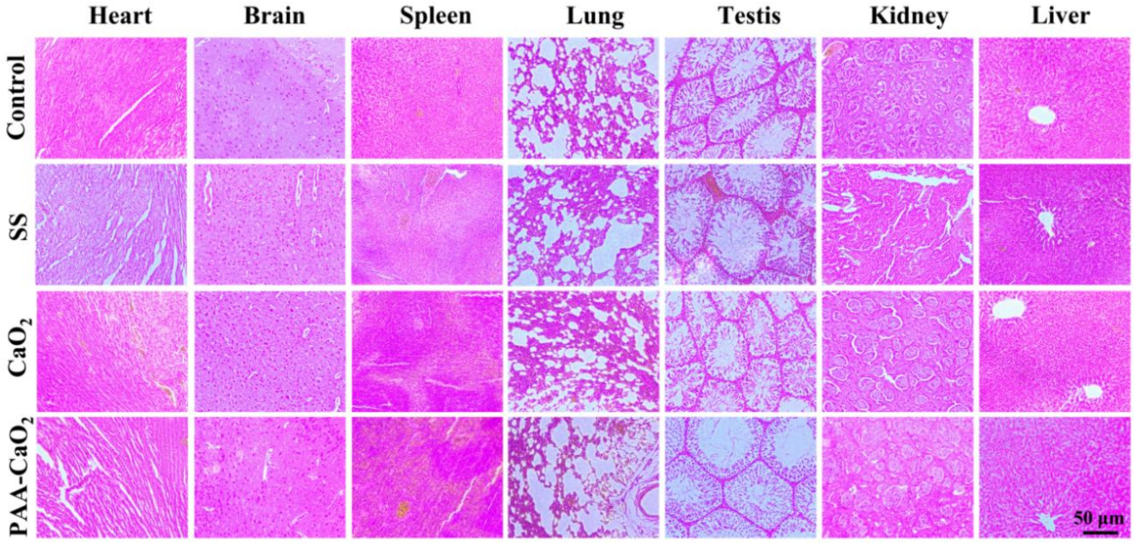

Fig.S9 Major organs histological observation of H&E staining in rats after treatment with different formulas.

Materials	Forms	Method	Effects	Year	References
FVEC	Hydrogel	Chemical crosslinking	Enhance the epithelium regeneration and scavenge ROS	2021	[13]
GS-CuO_2	Sponge	Chemical crosslinking	Release •OH and stimulate angiogenesis and collagen deposition	2021	[15]
Ag-AV-SF	Hydrogel	Photocrosslink	Promote the cell proliferation, migration and wound healing	2021	[19]
CuO_2	Nanoparticles	Chemical crosslinking	Accelerate wound healing in bacteria infected diabetic ulcer	2021	[21]
CSG-PEG/DMA/Zn	Hydrogel	Photo-polymerization	Antibacterial, antioxidant,and hemostatic	2022	[26]
EGF@CCN	Nanoparticles	Ion crosslinking	Promote wound healing	2021	[38]
PAA/PAH	Electrospun Fiber	Chemical crosslinking and electrospun	Mimic the extracellular matrix morphology and antioxidant	2021	[39]
PCL/VMT	Scaffold	Ion-exchange method and electrospun	Promote the wound healing in diabetic wound	2022	[55]
PDA-NPs@MSC-sEV	Hydrogel	Chemical crosslinking	Promote radiation combined with skin wound healing	2023	[57]
PAA-CaO_2	Nanoparticles	Chemical crosslinking	Antibacterial, reinforce angiogenesis, promte cell proliferation/migration	2023	This work

Table.S1 The comparison of PAA-CaO_2 nanoparticles' forms, methods, and effects with other biomaterials used in wound therapy.

Statistical analysis

The experiments were repeated at least 3 times, and the data was represented as average ± standard deviation (SD). The comparison between the two groups used unpaired student's T-test, and the comparison between multiple groups used a single

factor variance analysis (One-Way analysis of Variance, ANOVA). The difference is statistically significant with an asterisk (ns: non-significant, * $P < 0.05$, ** $P < 0.01$, *** $P < 0.001$, **** $P < 0.0001$). Statistical analysis was conducted using the GraphPad Prism software, and $P < 0.05$ was considered statistically significant.

Acknowledgments

This work was supported by grants from the Natural Science Foundation of Chongqing (cstc2020jcyj-msxmX0330, cstc2021jsyj-yzysbA0057); the National Natural Science Foundation of China (31971242, 12032007); the Science and Technology Innovation Project of Jinfeng Laboratory, Chongqing, China(jfkyjf202203001); the Project of Tutorial System of Medical Undergraduate in Lab Teaching & Management Center in Chongqing Medical University (LTMCMTS202107). We are also thankful for the First Batch of Key Disciplines on Public Health in Chongqing and the Public Experiment Center of State Bioindustrial Base (Chongqing), China.
