# Supplementary material for: Sprayed PAA-CaO2 nanoparticles combined with calcium ions and reactive oxygen species for antibacterial and wound healing
Source: Regen Biomater. 2023 Aug 21;10:rbad071. doi: 10.1093/rb/rbad071 (PMC10503269; doi:10.1093/rb/rbad071)
Supplement: rbad071_Supplementary_Data [file rbad071_supplementary_data.zip › Contributor (RB-2023-079-R2).pdf]

Author	Contributor
Hong Yu	Conceptualization, Data curation, Investigation, Methodology, Visualization, Writing-original draft
Jiale Sun	Investigation, Methodology, Writing-review & editing
Kepeng She	Investigation， Methodology, Writing-review & editing
Mingqi lv	Funding acquisition
Yiqiao Zhang	Methodology, Writing-original draft
Yawen Xiao	Investigation, Methodology
Yangkun Liu	Investigation, Methodology
Changhao Han	Investigation, Writing-review & editing
Xinyue Xu	Writing-review & editing
Shuqing Yang	Investigation, Methodology
Guixue Wang	Funding acquisition, Project administration
Guangchao Zang	Conceptualization, Funding acquisition, Project administration, Writing-review & editing
